# Photodynamic gel-bombs enhance tumor penetration and downstream synergistic therapies

**DOI:** 10.1038/s41392-025-02186-y

**Published:** 2025-03-19

**Authors:** Xiaole Bai, Fanliang Meng, Xuejiao Wang, Linyun He, Chao Fan, Liangjie Tian, Yangning Zhang, Jiahao Pan, Qun Wu, Xiangrong Hao, Ying Wang, Bo-Feng Zhu, Jun-Bing Fan, Bin Cong

**Affiliations:** 1 Guangzhou Key Laboratory of Forensic Multi-Omics for Precision Identification, School of Forensic Medicine, Southern Medical University, 510515 Guangzhou, P.R. China; 2 Cancer Research Institute, Experimental Education/Administration Center, School of Basic Medical Sciences, Southern Medical University, 510515 Guangzhou, P.R. China; 3 Department of Obstetrics and Gynecology, Nanfang Hospital, Southern Medical University, 510515 Guangzhou, P.R. China; 4 Department of Breast Surgery, Zhujiang Hospital, Southern Medical University, 510282 Guangzhou, P.R. China; 5 Shenzhen Key Laboratory of Viral Oncology, The Clinical Innovation & Research Center (CIRC), Shenzhen Hospital, Southern Medical University, 518101 Shenzhen, P.R. China; 6 Key Laboratory of Forensic Medicine in Shanxi Province, School of Forensic Medicine, Shanxi Medical University, 030600 Jinzhong, P.R. China; 7 Hebei Key Laboratory of Forensic Medicine, Collaborative Innovation Center of Forensic Medical Molecular Identification, College of Forensic Medicine, Hebei Medical University, 050017 Shijiazhuang, P.R. China

**Keywords:** Drug delivery, Drug development

## Abstract

Nanoparticle-based drug delivery system remains a significant challenge in the current treatment of solid tumors, primarily due to their limited penetration capabilities. Herein, we successfully engineer photodynamic gel-bombs (DCM@OPR) capable of penetrating deeply into tumor tissues utilizing the photodynamic-triggered explosive energy and receptor-mediated transcytosis, significantly enhancing the therapeutic efficacy of breast cancer. The photodynamic gel-bombs were fabricated by loading powerful components of chlorin e6 and MnO_2_ nanoparticles, as well as Doxorubicin, into a crosslinked Ca^2+^-gel. Upon exposure to laser irradiation, the obtained photodynamic gel-bombs are capable of generating explosive energy, resulting in their fragmentation into numerous nanofragments. The photodynamic-triggered explosive energy subsequently drives these nanofragments to deeply penetrate into tumor tissues through gap leakage among tumor cells. In addition, the photodynamic-triggered explosive energy also promotes the escape of those therapeutic components (including chlorin e6, MnO_2_ nanoparticles, and doxorubicin) and nanofragments from lysosomes. In the subsequent stages, these nanofragments also exhibit excellent transcytosis capacity, facilitating deep penetration into tumor tissues. As expected, the enhanced penetration and accumulation of therapeutic components into tumor tissues can be achieved, significantly enhancing the anti-proliferation capacity against breast cancer.

## Introduction

Tumors persistently represent a substantial risk to global human health. Over recent decades, there has been considerable interest in the development of nanoparticle-based drug delivery systems as they hold promise for enhancing anti-tumor efficacy.^1–4^ However, these systems present substantial challenges in treating solid tumors.^5,6^ This is primarily due to their limited penetration capabilities, which are hindered by the abnormal tumor microenvironment,^7^ such as dense stromal cells and extracellular matrix,^8–10^ abnormal tumor vascular network,^11,12^ and increased interstitial fluid pressure.^13–15^

Numerous efforts have been undertaken to enhance the penetration capability of nanodrugs into tumor tissues to enhance their anti-tumor efficacy. These strategies usually involved remodeling the tumor microenvironment by utilizing the distinctive characteristics of the tumor microenvironment,^16–18^ such as its low pH,^19–21^ hypoxia^22,23^ and specific enzymes.^24,25^ A variety of responsive drug delivery systems have been attempted to improve their penetration capacities. In particular, breakable nanoparticle-based drug delivery systems, have received significant effectiveness in improving penetration. These breakable nanoparticles typically exhibit large sizes in circulation but fragment into smaller ones in response to stimuli within the tumor microenvironment.^22,23,26^ Despite attempts to increase the penetration depth of these breakable nanoparticle-based drug delivery systems through size reduction, their penetration still relies on passive diffusion through the dense paracellular matrix without a driving force, thereby resulting in slow and inefficient penetration. Therefore, the effectiveness of these systems remains constrained due to their moderate response efficiency.

**Fig. 1:**
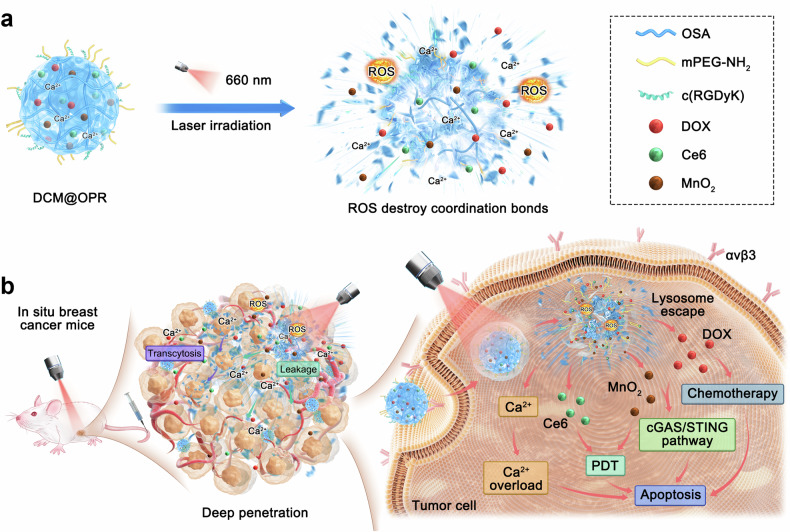
Schematic illustration of photodynamic gel-bombs for enhanced tumor deep penetration and synergistic therapies. **a** Schematic illustration of the blast of photodynamic gel-bombs upon laser irradiation. Upon exposure to laser irradiation of solid tumors, the obtained photodynamic gel-bombs could generate a substantial quantity of ROS to provide sufficient explosive energy, enabling them to burst into a large number of nanofragments. **b** Schematic illustration of deep penetration and synergistic therapies of the tumor. During this process, the photodynamic-triggered explosive energy could propel those nanofragments to penetrate deeply into tumor tissues through gap leakage among tumor cells. Subsequently, internalized nanofragments demonstrated excellent transcytosis capacity. On the other hand, when some photodynamic gel-bombs were internalized into tumor cells, the photodynamic-triggered explosive energy could also disrupt intracellular lysosome membranes, which in turn facilitated the escape of therapeutic components such as Ce6, MnO_2_ nanoparticles, DOX, and Ca^2+^ from lysosomes, allowing an efficient accumulation of them within the tumor cells. Consequently, the enhanced anti-proliferative capacity against solid tumors was achieved through photodynamic gel-bomb-mediated synergistic therapies

Herein, photodynamic gel-bombs were demonstrated, enabling to efficiently penetrate into tumor tissues by the photodynamic-triggered explosive energy driving force and receptor-mediated transcytosis, thereby significantly enhancing the therapeutic efficacy for breast cancer. As shown in Fig. 1 and Supplementary Fig. 1, the photodynamic gel-bombs were constructed by loading powerful components chlorin e6 (Ce6) and manganese dioxide (MnO_2_) nanoparticles along with the chemotherapeutic Doxorubicin (DOX) into a calcium ion (Ca^2+^) crosslinked alginate gel. When the breast cancer site was exposed to laser irradiation, the photodynamic gel-bombs, once they had traveled into the tumor site, were able to generate sufficient reactive oxygen species (ROS) to provide explosive energy. Therefore, the first driving force for the photodynamic gel-bombs penetration was photodynamic-triggered explosive energy. Upon exposure to laser irradiation, these photodynamic gel-bombs enabled to disintegrate gel networks into numerous nanofragments. Meanwhile, this explosive energy further drove these nanofragments deep penetration into the tumor tissues through gap leakage among tumor cells. The second driving force for the photodynamic gel-bombs penetration was receptor-mediated transcytosis. When photodynamic gel-bombs were internalized into tumor cells, the explosive energy could also disrupt intracellular lysosome membranes, thereby facilitating the escape of nanofragments from lysosomes. In the subsequent stages, these nanofragments demonstrated excellent transcytosis capacity, allowing for effective accumulation and positive deep penetration in tumor tissues, which was beneficial to enhance their anti-tumor activity. Beyond Ce6, MnO_2_ nanoparticles utilized H_2_O_2_ and H^+^ in the tumor microenvironment to produce O_2_, which could also augment the photodynamic efficiency.^27^ Meanwhile, the resulting Mn^2+^ could also synergistically combine with DOX to co-stimulate the enhancement of the cGAS/STING pathway, thereby conferring potential anti-tumor properties. Additionally, the released excessive Ca^2+^ enabled to further induce tumor cell apoptosis.^28–30^ Consequently, the enhanced penetration and accumulation of therapeutic components into tumor tissues significantly improved the treatment efficacy for tumors through photodynamic gel-bomb-mediated synergistic therapies.

## Results

### Synthesis and characterization of the photodynamic gel-bombs (DCM@OPR)

**Fig. 2:**
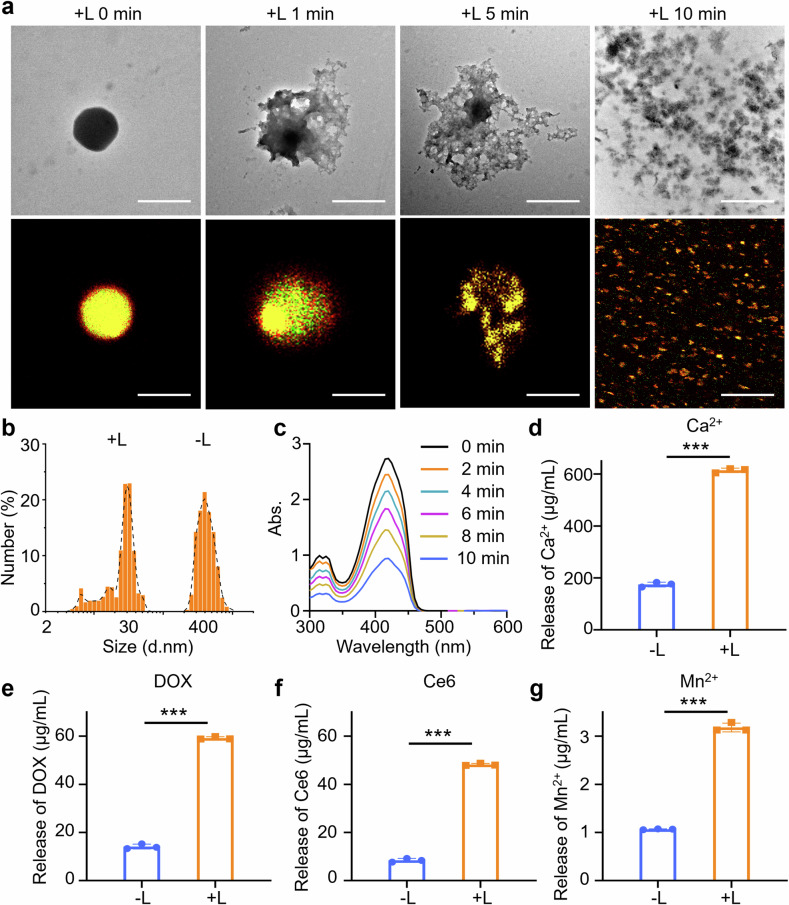
Synthesis of characterization of photodynamic gel-bombs (DCM@OPR). **a** TEM images and CLSM images of photodynamic gel-bombs before and after laser irradiation at different times. Scale bars: 200 nm in TEM images, 400 nm in CLSM images. The results indicated that upon exposure to laser irradiation, the obtained photodynamic gel-bombs could burst into a large number of nanofragments. **b** The size of photodynamic gel-bombs (DCM@OPR) and DCM@OPR nanofragments measured by DLS. **c** UV absorption spectra of DPBF mixed with photodynamic gel-bombs followed by laser irradiation for different times. **d–g** The release of Ca^2+^, DOX, Ce6, and Mn^2+^ within the photodynamic gel-bombs before and after laser irradiation. These results demonstrated that upon exposure to laser irradiation, the photodynamic gel-bombs could lead to a significant release of Ca^2+^, DOX, Ce6, and Mn^2+^. “−L” represented “without laser irradiation”, and “+L” represented “with laser irradiation”. Data are presented as mean ± SD. *n* = 3 per group. *p < 0.05, **p < 0.01, ***p < 0.001

The photodynamic gel-bombs were fabricated by loading Ce6 and MnO_2_ nanoparticles as well as chemotherapeutic DOX into a Ca^2+^ crosslinked alginate gel (Supplementary Fig. 1). In a typical procedure, sodium alginate was first modified with mPEG-NH_2_ and c(RGDyk) to produce mPEG-OSA-c(RGDyK) (OPR). Subsequently, the photodynamic gel-bombs (DCM@OPR) were synthesized by co-incorporating Ce6, MnO_2_ nanoparticles (Supplementary Fig. 2) and DOX into OPR, followed by Ca^2+^ crosslinking under high-speed shearing. c(RGDyk), an integrin with high binding affinity to αvβ3 (overexpressed by the neovascular endothelial cells and tumor cells, such as 4T1 cells),^31,32^ would endow the obtained photodynamic gel-bombs (DCM@OPR) with good tumor targeting ability. Owing to enhance the tumor-targeting ability, the accumulation and internalization of the obtained DCM@OPR within tumor tissues and tumor cells would increase accordingly, which is beneficial for them to proceed with subsequent positive penetration into tumor tissues. Transmission electron microscopy (TEM) images revealed that DCM@OPR exhibited a spherical shape (Fig. 2a) and dynamic light scattering (DLS) indicated that their hydration diameters were 426.29 ± 31.10 nm (Fig. 2b). Additional, DLS results revealed that the hydration diameters of DCM@OPR, Cy5-DCM@OPR, and Cy7-DCM@OPR were 426.29 ± 31.10, 477.58 ± 27.69, and 464.04 ± 35.81 nm, respectively (Supplementary Fig. 3). While, TEM images showed the sizes of DCM@OPR were approximately shrunk in half after dehydration (Fig. 2a, Supplementary Fig. 4). As shown in Supplementary Fig. 5, confocal laser scanning microscope (CLSM) images suggested that DOX and Ce6 were uniformly distributed within DCM@OPR. UV–vis spectra showed that DCM@OPR had characteristic absorption peaks at 490 nm (similar to DOX) and at 409 and 669 nm (similar to Ce6) (Supplementary Fig. 6). Additionally, energy dispersive X-ray spectroscopy (EDS) analysis detected characteristic peaks of Mn^2+^ (Supplementary Fig. 7). These results indicated the successful loading of Ce6, DOX and MnO_2_ nanoparticles into the photodynamic gel-bombs. The encapsulation efficiency (EE%) and loading efficiency (LE%) of DOX, Ce6 and MnO_2_ within DCM@OPR were shown in Supplementary Table. 1. The zeta potential of DCM@OPR were measured to be −34.36 ± 2.14 mV (Supplementary Fig. 8). Furthermore, the structural stability of DCM@OPR in 10% fetal bovine serum (FBS) was evaluated, and the results showed that there were no significant changes in their sizes and polymer dispersity index (PDI) within 168 h (Supplementary Fig. 9), suggesting good structural stability.

We first investigated the photodynamic effectiveness of the obtained DCM@OPR upon exposure to laser irradiation. Interestingly, upon exposure to laser irradiation, the DCM@OPR exhibited bomb-like properties, which gradually expanded and then burst into a large number of nanofragments accompanying the extension of laser irradiation time (Fig. 2a and Supplementary Fig. 5). DLS results indicated a significant reduction in the size of DCM@OPR following laser irradiation, suggesting their disintegration. The size distribution of the obtained DCM@OPR nanofragments was identified, predominantly measuring approximately 26.91 nm in size (Fig. 2b, Supplementary Fig. 10). Additionally, the hydration diameters of DCM@OPR nanofragments, Cy5-DCM@OPR nanofragments and Cy7-DCM@OPR nanofragments were predominantly around 26.91, 31.42, and 31.22 nm, respectively (Supplementary Fig. 11).

To explore the burst mechanism of photodynamic gel-bombs, we proposed that ROS produced through photodynamic Ce6 may provide explosive energy to disintegrate the existing networks of the gel. We used a 1,3-diphenylisobenzofuran (DPBF) probe to analyze the production of ROS within the photodynamic gel-bombs at different laser irradiation times. The results demonstrated that the synthesized photodynamic gel bomb was capable of generating a substantial quantity of ROS upon exposure to laser irradiation. There was a positive correlation observed between the duration of irradiation and the amount of ROS produced; an increase in irradiation time led to an enhanced generation of ROS (Fig. 2c). Then, we compared the energy required to break the coordination bond between Ca^2+^ and alginate (*E*_1_) and the energy released from ROS emission under laser exposure (*E*_2_). To facilitate the calculation, we hypothesized that 1 mol of Ca^2+^ was completely crosslinked to form the photodynamic gel-bombs. According to the loading amount of Ce6 within the photodynamic gel-bombs, the amount of substance of Ce6 was calculated to be 0.05 mol. The Gibbs free energy of the photodynamic gel-bombs (coordination compound system) could be calculated by the following equation^33^:

1 $${{E}}_{1}=\Delta {G}=-{RT\; {\rm {log}}}K$$

where Δ*G* was the Gibbs free energy, *R* was the ideal gas constant, *T* was the temperature, and *K* was the complexation constant between Ca^2+^ and alginate. The calculation formula for *K*^34^ was

2 $${K}\,=\,\frac{{{C}}_{1}}{{{C}}_{2}\,\times \,{{{C}}_{3}}^{{n}}}$$

*C*_1_, *C*_2_, and *C*_3_ represented the concentrations of calcium alginate, Ca^2+^, and alginate after reaching complexation equilibrium, respectively. Through experimentation and calculation, *K* was calculated to be 2.97 × 10^5^. Thus, the Δ*G* required to break these coordination bonds was calculated to be Δ*G* = 5822 J (*E*_1_).

Since 1 mol of Ce6 produced at least 0.75 mol of ROS.^35^ The energy that can be produced by Ce6 in the photodynamic gel-bombs (*E*_2_) could be calculated by the following equation^35–37^:

3 $${{E}}_{2}={{n}}_{{\rm{Ce}}6}\times 0.75\times {E}_{\rm{ROS}}$$

where *n*_Ce6_ was the amount of substance of Ce6 that can be loaded in the photodynamic gel-bombs. *E*_ROS_ was the energy produced by 1 mol ROS. 1 mol ROS could release approximately 2.70 mol eV energy during oxidation reactions,^36,37^ which corresponds to 263.0 kJ of energy. Therefore, the energy generated by Ce6 within the photodynamic gel-bombs was calculated to be *E*_2_ = 9862 J. The result indicated that upon exposure to laser irradiation, the abundant photodynamic-triggered explosive energy (*E*_2_) was much larger than the energy required to break these coordination bonds (*E*_1_). Thus, the photodynamic-triggered explosive energy was sufficient to disrupt the coordination bonds between Ca^2+^ and alginate (*E*_2_ ＞ *E*_1_), leading to the burst of the photodynamic gel-bombs.

To verify whether the photodynamic gel-bombs were disintegrated into single molecules, such as OSA or OSA-mPEG (OP), we conducted a thorough examination of the nanofragments produced by DCM@OPR gel-bombs after laser irradiation. This included an analysis of their molecular weight, structural formula, and the colocalization of DOX and Ce6 within these nanofragments using CLSM, DLS for size distribution and TEM imaging. The molecular weight results indicated that the number-average molecular weight of DCM@OPR nanofragments and DCM@OPR gel-bombs was 16362 and 34922, respectively, indicating the disintegration of DCM@OPR gel networks (Supplementary Table. 2). The results from the FTIR spectra and ^1^H NMR suggested that DCM@OPR gel-bombs and DCM@OPR nanofragments were highly consistent (Supplementary Figs. 12, 13). And similar to the DCM@OPR gel-bombs, the characteristic peaks of OSA, mPEG-NH_2_ and c(RGDyk) persisted in the DCM@OPR nanofragments in the ^1^H NMR results (Supplementary Figs. 13, 14). Moreover, CLSM results also indicated the colocalization of DOX and Ce6 within the DCM@OPR nanofragments (Fig. 2a, Supplementary Fig. 5). At the same time, TEM images also demonstrated that DCM@OPR gel-bombs break-up into a large number of nanofragments (Fig. 2a). On the other hand, we assumed that if the DCM@OPR gel-bombs broke-up into single molecules (OSA or OSA-mPEG), which could not be detected by DLS due to OSA or OSA-mPEG was highly water-solubility (Supplementary Fig. 10). While, the size distribution of DCM@OPR nanofragments could be identified (Fig. 2b). These results indicated that the DCM@OPR gel-bombs may proceed with an incomplete disintegration, and the obtained DCM@OPR nanofragments remained crosslinked gel networks. Further, EE% and drug release of DCM@OPR nanofragments were identified. The EE% of DOX, Ce6, and MnO_2_ within nanofragments were shown in Supplementary Table 3. The results of the drug release profile indicated that the DCM@OPR nanofragments demonstrated effective drug release (Supplementary Fig. 15). Moreover, the process of exposure to laser irradiation, accompanied by a significant release of therapeutic components, including DOX, Ce6, MnO_2_ nanoparticles and Ca^2+^ (Fig. 2d–g), which was beneficial to promoting deep penetration and accumulation of these therapeutic components within tumor tissues.

### The biological activities of the photodynamic gel-bombs (DCM@OPR) in vitro

**Fig. 3:**
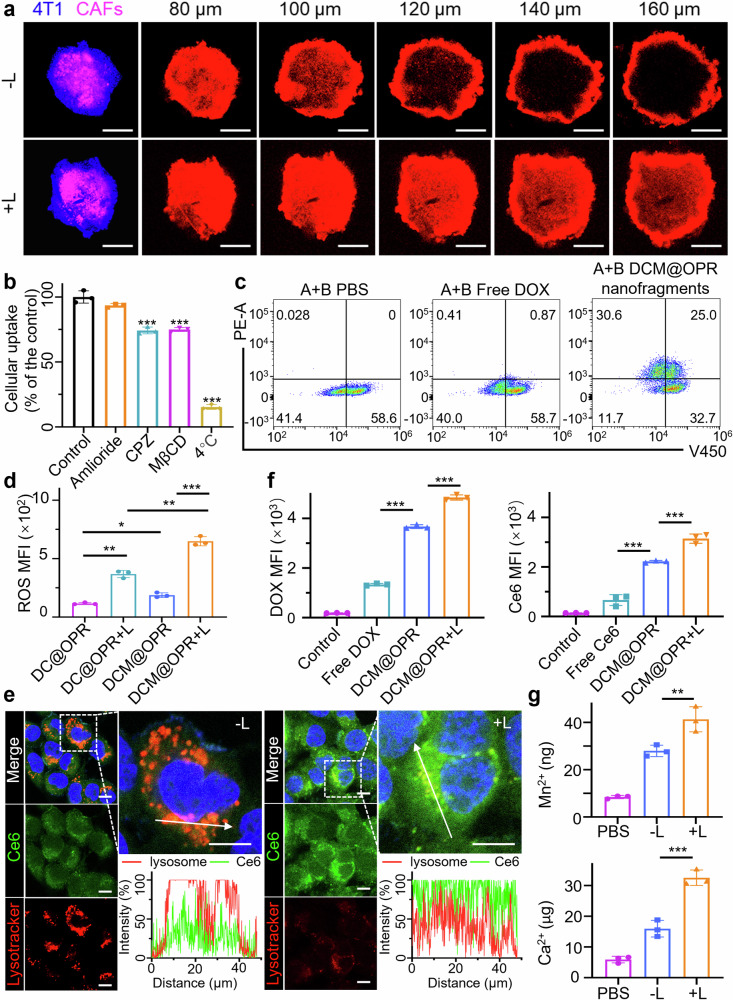
The deep penetration and lysosomal escape of photodynamic gel-bombs (DCM@OPR) in vitro. **a** Deep penetration capacity of DCM@OPR in heterospheroids before and after laser irradiation. Scale bars: 100 μm. The results indicated that the penetration capabilities of these photodynamic gel-bombs were enhanced upon exposure to laser irradiation. **b** The endocytic pathway of DCM@OPR nanofragments. Data are presented as mean ± SD. *n* = 3 per group. **c** The transcytosis efficiency of free DOX, DCM@OPR nanofragments from 4T1 cells to 4T1 cells determined by PE-A^+^/V450^+^ of flow cytometry. DOX detection via PE-A channel, Hoechst detection via V450 channel. A cells: 4T1 cells, B cells: Hochest33342-stained 4T1 cells. The subpopulation cells of PE-A^+^/V450^+^ represented transcytosis efficiency. **d** The quantitative analysis of the generation of ROS by these photodynamic gel-bombs under hypoxic conditions. The results indicated that the photodynamic gel-bombs (DCM@OPR) exhibited the best ROS production upon exposure to laser irradiation. Data are presented as mean ± SD. *n* = 3 per group. **e** Co-localization of CM@OPR with lysosomes using CLSM before and after laser irradiation. Scale bars: 10 μm. **f** Cellular accumulation of DOX and Ce6 in 4T1 cells using flow cytometry. DOX detection via PE-A channel, Ce6 detection via FITC channel. Data are presented as mean ± SD. *n* = 3 per group. **g** Cellular accumulation of Mn^2+^ and Ca^2+^ in 4T1 cells using ICP-MS. Data are presented as mean ± SD. *n* = 3 per group. *p < 0.05, **p < 0.01, ***p < 0.001

We investigated the in vitro biological activities of the photodynamic gel-bombs (DCM@OPR), including deep penetration, cellular uptake, endocytosis pathway, transcytosis efficiency, ROS production, lysosomal escape, intracellular drug accumulation, and cytotoxicity. First, multicellular tumor spheroid model was constructed utilizing 4T1 cells to evaluate the deep penetration capability of free DOX, free Ce6, and free Cy5-OPR with the same amount of DOX, Ce6, and Cy5 without laser irradiation. The results indicated that the penetration capacity of free Ce6 was similar to that of free DOX. However, the penetration ability of both free Ce6 and free DOX was inferior to that of free Cy5-OPR in 4T1 multicellular tumor spheroids (Supplementary Fig. 16). Furthermore, we also analyzed the penetration abilities of free DOX, D@OP (DOX incorporated into the gel and without c(RGDyk) modification) and D@OPR (DOX incorporated into the gel and with c(RGDyk) modification) under the same experimental conditions. The results indicated that the penetration capabilities of D@OP and D@OPR were higher than the free DOX. Notably, D@OPR outperformed D@OP due to its good accumulation and internalization in tumor tissues and tumor cells as a result of superior targeting capacity conferred by c(RGDyk) (Supplementary Fig. 17). Further, heterospheroids (co-cultures of cancer-associated fibroblasts (CAFs) and 4T1 cells) were constructed to assess the penetration ability of the DCM@OPR with or without laser irradiation. The DCM@OPR + L group (with laser irradiation) exhibited an enhanced penetration into heterospheroids compared to the DCM@OPR group (without laser irradiation) (Fig. 3a). This was demonstrated by brighter fluorescence extending from the periphery to more central areas within the heterospheroids. Furthermore, fluorescence co-localization images suggested an overall increase in tumor spheroid brightness for the DCM@OPR + L group compared to the DCM@OPR group (Supplementary Fig. 18). Furthermore, to validate the concept of explosive energy-driven deep penetration, we compared the penetration capabilities of pre-collapsed DCM@OPR nanofragments and DCM@OPR + L in heterospheroids. The results clearly indicated that DCM@OPR + L exhibited a significantly higher capacity for deep penetration into heterospheroids compared to DCM@OPR nanofragments (Supplementary Fig. 19).

Because the photodynamic gel-bombs (DCM@OPR) were disintegrated into nanofragments after laser irradiation, we next investigated their cellular uptake and endocytosis pathway. The results indicated that the cellular uptake of DCM@OPR nanofragments was superior to free DOX. Upon exposure to laser irradiation, the cellular uptake of DCM@OPR nanofragments enhanced in comparison with that of them without laser irradiation (Supplementary Fig. 20). Subsequently, we also investigated the endocytosis pathway of DCM@OPR nanofragments. The endocytosis inhibition experiment results indicated that the nanofragments mainly proceeded with clathrin-mediated endocytosis and caveolin-mediated endocytosis (Fig. 3b). Previous experimental results show that transcytosis is primarily associated with caveolin-mediated endocytosis^38^; therefore, we studied the transcytosis ability of the nanofragments using flow cytometry. The results demonstrated that the nanofragments exhibited enhanced transcytosis compared to free DOX in 4T1 cells, allowing effective accumulation and deep penetration in tumor tissues (Fig. 3c).

The generated ROS are the key factors that induce cytotoxicity in photodynamic therapy (PDT). We next explored how DCM@OPR affected ROS production within 4T1 cells under hypoxic conditions. The results indicated that the ROS production in the DCM@OPR group was higher than that in the DC@OPR group (DOX and Ce6 incorporated into the gel) under the same conditions. Significantly, upon exposure to laser irradiation, the ROS production was increased in comparison with the non-irradiated group. In particular, DCM@OPR exhibited the best ROS production when exposed to laser irradiation (Fig. 3d and Supplementary Fig. 21). This was because ROS generation in PDT required laser irradiation and oxygen in addition to photosensitizers.^39,40^ The MnO_2_ nanoparticles present within the photodynamic gel-bombs could exploit the acidic microenvironment of tumor cell metabolism to generate additional oxygen in situ, alleviating hypoxia and promoting ROS generation.^41,42^

When particles are internalized into tumor cells, a primary biological obstacle is lysosomes that enable to degrade these particles.^43,44^ We investigated the fate of the photodynamic gel-bombs after they transferred into lysosomes. In our study, to avoid the conflict of fluorescent channels, only Ce6 and MnO_2_ nanoparticles were incorporated into the gel (referred to as CM@OPR) in the absence of DOX. The results indicated that post-laser irradiation, a significant dimming was observed in the red fluorescence signal associated with lysosomes, suggesting a potential damage of lysosome membranes. Conversely, the green fluorescence signal from Ce6 within the cytoplasm experienced an enhancement, indicating that the content of Ce6 was elevated in the cytoplasm. The colocalization fluorescence intensity also revealed a decrease in the red lysosomal fluorescence and an increase in the green Ce6 fluorescence post-laser exposure (Fig. 3e). In addition, we performed an acridine orange (AO) assay to further strengthen and confirm the occurrence of lysosomal escape. As illustrated in Supplementary Fig. 22, prior to laser irradiation, AO displayed red fluorescence within the acidic lysosomes, indicating an intact lysosomal membrane. Following laser irradiation, AO exhibited green fluorescence in the neutral or slightly alkaline cytoplasm, implying that the lysosomal membrane had been disrupted and AO had escaped from the acidic lysosomal environment into the cytoplasm. This disruption of the lysosomal membrane following laser irradiation resulted in lysosomal escape.

Effective cellular internalization and drug accumulation were prerequisites for DCM@OPR to efficiently exert anti-tumor efficacy. Firstly, we investigated the cellular uptake of DCM@OPR in vitro. As expected, cellular internalization of DOX results demonstrated that compared to the DCM@OPR group (without laser irradiation), DCM@OPR + L group (with laser irradiation) exhibited significantly stronger red fluorescence signals, indicating a higher intracellular accumulation of therapeutic component (Supplementary Fig. 23). Subsequently, we quantified the accumulation of therapeutic components (including DOX, Ce6, Mn^2+^, and Ca^2+^) within 4T1 cells. The presence of DOX and Ce6 was detected by flow cytometry using PE-A and FITC channels, respectively, while Mn^2+^ and Ca^2+^ were measured by inductively coupled plasma-mass spectrometry (ICP-MS). The results revealed that the accumulation levels of DOX, Ce6, Mn^2+^, and Ca^2+^ in the DCM@OPR + L group (with laser irradiation) were significantly higher than those in the DCM@OPR group (without laser irradiation) (Fig. 3f, g). The enhanced intracellular accumulation of therapeutic components would be attributed to the improved lysosome escape under laser irradiation. Upon exposure to laser irradiation, the photodynamic-triggered explosive energy was capable of damaging the lysosomal membrane. This allowed the escape of therapeutic components and DCM@OPR nanofragments from lysosomes, thereby avoiding degradation by acids and enzymes within the lysosomes, and consequently enhancing intracellular accumulation.

The cytotoxicity of DCM@OPR on tumor cells was evaluated. The antiproliferative effects of free DOX, D@OPR (only DOX incorporated into the gel), DC@OPR (DOX and Ce6 incorporated into the gel), DC@OPR + L (DOX and Ce6 incorporated into the gel and with laser irradiation), DCM@OPR (DOX, Ce6 and MnO_2_ nanoparticles incorporated into the gel) and DCM@OPR + L (DOX, Ce6 and MnO_2_ nanoparticles incorporated into the gel and with laser irradiation) on 4T1 cells were investigated using 3-(4,5-dimethylthiazol)-2,5-diphenyl-tetrazolium bromide (MTT) assay (Supplementary Fig. 24). All treatment groups demonstrated dose-dependent inhibitory effects. The results demonstrated that upon laser irradiation, a significant antiproliferative effect could be observed in the photodynamic groups (DC@OPR + L and DCM@OPR + L). Compared to the DC@OPR + L group, the DCM@OPR + L group exhibited the most potent antiproliferative effects. The enhanced antiproliferative effects of the DCM@OPR + L group should be attributed to the presence of MnO_2_ nanoparticles improving the efficacy of PDT. Additionally, a significant release of Ca^2+^ from DCM@OPR + L group could also be detected, which could disrupt cellular Ca^2+^ homeostasis to cause cell apoptosis.^28–30^ In addition, we also validated the antiproliferative effects of DCM@OPR in MDA-MB-231 cells. The results similarly indicated that DCM@OPR and DCM@OPR + L groups showed stronger cytotoxicity compared to the existing drug DOX. Notably, the DCM@OPR + L group exhibited the strongest cytotoxic effect (Supplementary Fig. 25).

To investigate the synergistic effect within the photodynamic gel-bombs, we conducted in vitro cytotoxicity assays to determine the drug concentrations that achieve half-maximal inhibitory concentration (IC_50_) for both individual and combined chemotherapy/PDT. Following this, we computed the combination index (CI) to assess potential synergistic effects. A CI value of less than 1 suggests that the combined effect is greater than the sum of the individual effects, which is defined as synergism. Conversely, a CI value of more than 1 indicates that the combined effect is less than the sum of the individual effects, referred to as antagonism.

4 $$\text{CI}=\frac{\text{IC}_{50}\left(\text{DCM@OPR}+\text{L}\right)}{\text{IC}_{50}\left(\text{D@OPR}\right)}+\frac{\text{IC}_{50}\left(\text{DCM@OPR}+\text{L}\right)}{\text{IC}_{50}(\text{CM@OPR}+\text{L})}$$

According to formula (4), Supplementary Tables 4 and 5, the CI was calculated as 0.26, which is <1. This suggested a synergistic effect between DOX, Ce6 and MnO_2_ within the photodynamic gel-bombs. These results indicated that the photodynamic gel-bombs (DCM@OPR) could synergistically enhance the anti-proliferative activity.

### The biological activities of the photodynamic gel-bombs (DCM@OPR) in vivo

**Fig. 4:**
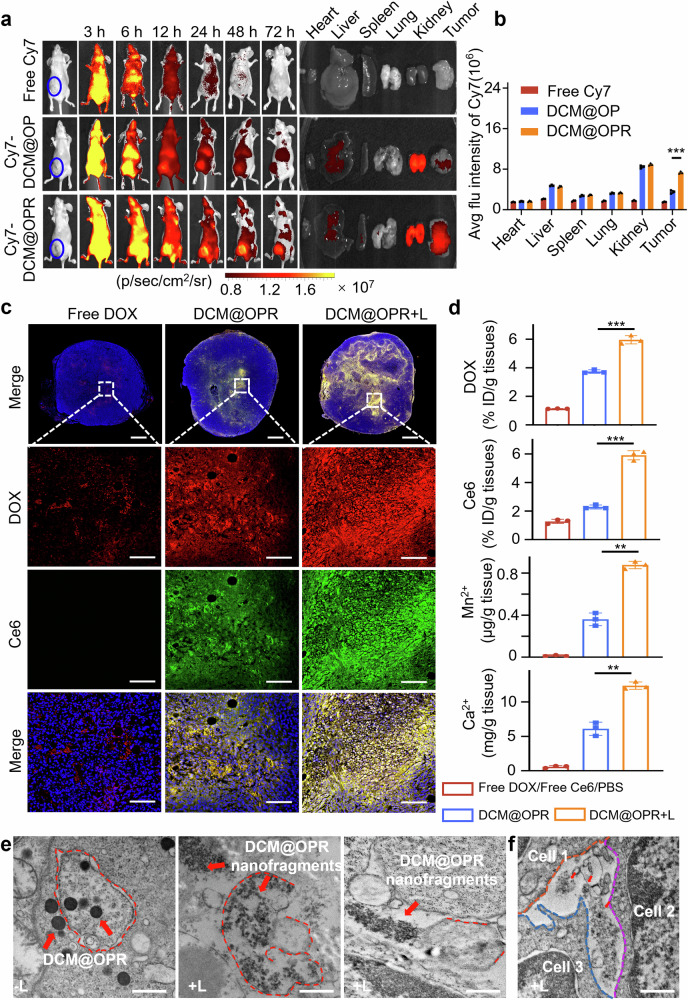
Biodistribution, deep penetration, therapeutic component accumulation and lysosomal escape of photodynamic gel-bombs (DCM@OPR) in vivo. **a** In vivo biodistribution of free Cy7, Cy7-DCM@OP, and Cy7-DCM@OPR at different times in 4T1 in situ tumor-bearing Balb/c nude mice after administration. **b** Average fluorescence intensity of ex vivo organs and tumors from different groups. *n* = 3 per group. **c** CLSM images of the 4T1 in situ tumor-bearing Balb/c mice complete tumor tissues. The scale bars of the upper line were 1000 μm, scale bars of the below three lines columns were 100 μm. **d** Concentrations of DOX, Ce6, Mn^2+^, and Ca^2+^ in tumors of 4T1 in situ tumor-bearing Balb/c mice. *n* = 3 per group. These results indicated that upon exposure to laser irradiation, DCM@OPR could deeply penetrate into tumor tissues, allowing more therapeutic components to accumulate in tumor tissues. **e**, **f** TEM images of the distribution of DCM@OPR in 4T1 tumor tissues with or without laser irradiation. Upon exposure to laser irradiation, DCM@OPR experienced a photodynamic-triggered explosion, resulting in the formation of nanofragments. This explosive energy could damage the lysosomal membrane, thereby enabling nanofragments to escape from lysosomes into other cellular regions. Besides, except for intracellular, these nanofragments were also found to exist in the gap leakage among tumor cells, suggesting that the explosive energy may drive these nanofragments deep penetration through gap leakage among tumor cells. Scale bars: 500 nm. Data are presented as mean ± SD. *p < 0.05, **p < 0.01, ***p < 0.001

The biodistribution and tumor-targeting capacities of the photodynamic gel-bombs (DCM@OPR) were investigated in vivo. Cy7 fluorescence signals were detected at different times post-tail vein injection of free Cy7, Cy7-DCM@OP, and Cy7-DCM@OPR in 4T1 in situ tumor-bearing Balb/c nude mice. Mice were euthanized at intervals of 24, 48, and 72 h, respectively. Subsequently, major organs (including the heart, liver, spleen, lung, and kidney) and tumors were carefully excised to detect their accumulations. The results demonstrated that, compared to free Cy7 and Cy7-DCM@OP, the mice in the Cy7-DCM@OPR group exhibited the most potent in vivo fluorescence signals at the aforementioned time points. *In* ex vivo analyses of organs and tumors, the fluorescence signals of the Cy7-DCM@OP and Cy7-DCM@OPR groups were predominantly observed in metabolic organs (liver and kidney) and tumors. Compared to the free Cy7 and Cy7-DCM@OP, the Cy7-DCM@OPR group demonstrated the most fluorescence signals in tumor tissues, conferred by c(RGDyk), suggesting good targeting capacity (Fig. 4a, b, Supplementary Fig. 26).

Subsequently, the deep penetration of drugs into the tumor tissues was examined. As shown in Fig. 4c, compared to the free DOX group, the DCM@OPR group demonstrated more efficient deep penetration. This deep penetration was further amplified following laser irradiation. In the DCM@OPR + L group (with laser exposure), these therapeutic components (DOX and Ce6) were distributed from the boundary to the deep center of tumor tissues, suggesting a more efficient penetration in comparison with DCM@OPR (without laser exposure) and free DOX. Next, we performed fluorescent labeling of blood vessels to further assess the tissue penetration of DCM@OPR. As shown in Supplementary Fig. 27, at 1 h, a significant portion of DCM@OPR (Purple, marked with DOX) was observed within the tumor blood vessels (Cyan, labeled with CD34). As time progressed, DCM@OPR extravasated from the tumor blood vessels into the surrounding tumor tissues. The drug particles in the DCM@OPR + L group were found to be further away from the blood vessels compared to those in the DCM@OPR group, suggesting that DCM@OPR exhibited enhanced tumor penetration capacity under laser irradiation conditions. Furthermore, we evaluated the potential accumulation of DCM@OPR in the tumor microenvironment, specifically within tumor cells and macrophages. Supplementary Fig. 28 demonstrated that a higher concentration of DCM@OPR (Purple, denoted with DOX) was observed within tumor cells (Cyan, labeled with CD44) compared to macrophages (Yellow, labeled with F4/80). The quantity of these DCM@OPR within the tumor cells exhibited a gradual increase over time.

We subsequently detected the accumulation of DOX, Ce6, Mn^2+^, and Ca^2+^ in major organs and tumor tissues. The results demonstrated a significant increase in the concentrations of DOX, Ce6, Mn^2+^, and Ca^2+^ in tumor tissues following laser irradiation compared to their pre-irradiation levels (Fig. 4d and Supplementary Fig. 29). These results indicated that upon exposure to laser irradiation, DCM@OPR could deeply penetrate into tumor tissues, allowing more therapeutic components accumulation into tumor tissues, which is beneficial for enhancing anti-tumor efficacy.

Lysosomal escape phenomenon was also verified at the animal level. We used TEM to observe the escape of DCM@OPR from lysosomes in 4T1 tumor tissues before and after laser irradiation. Before laser exposure, a significant quantity of spherical DCM@OPR was observed to accumulate within the 4T1 tumor cells, into which the lysosomal membranes remained intact. However, following laser irradiation, these DCM@OPR experienced fragmentation, and meanwhile, this process resulted in a disruption of the lysosomal membranes, enabling these nanofragments to escape from lysosomes into the cytoplasm. As such, a significant number of DCM@OPR nanofragments were also observed in other cellular regions following laser irradiation, not just within the lysosomes (Fig. 4e, Supplementary Fig. 30). Besides, except for intracellular, we found that these nanofragments also existed in the gap leakage among tumor cells, suggesting that the explosive energy may drive these nanofragments deep penetration through gap leakage among tumor cells (Fig. 4f, Supplementary Fig. 31).

Additionally, we employed EDS to ascertain the alteration of elements within 4T1 tumor tissues pre and post-laser irradiation following treatment with DCM@OPR. The results suggested that, following treatment with DCM@OPR, in contrast to the levels of elements such as calcium and manganese present in 4T1 tumor tissues before laser irradiation, there was a notable elevation in these elements after laser irradiation, suggesting that laser irradiation may facilitate them release (Supplementary Fig. 32, Supplementary Table. 6).

### The anti-tumor activity of the photodynamic gel-bombs (DCM@OPR) in vivo

**Fig. 5:**
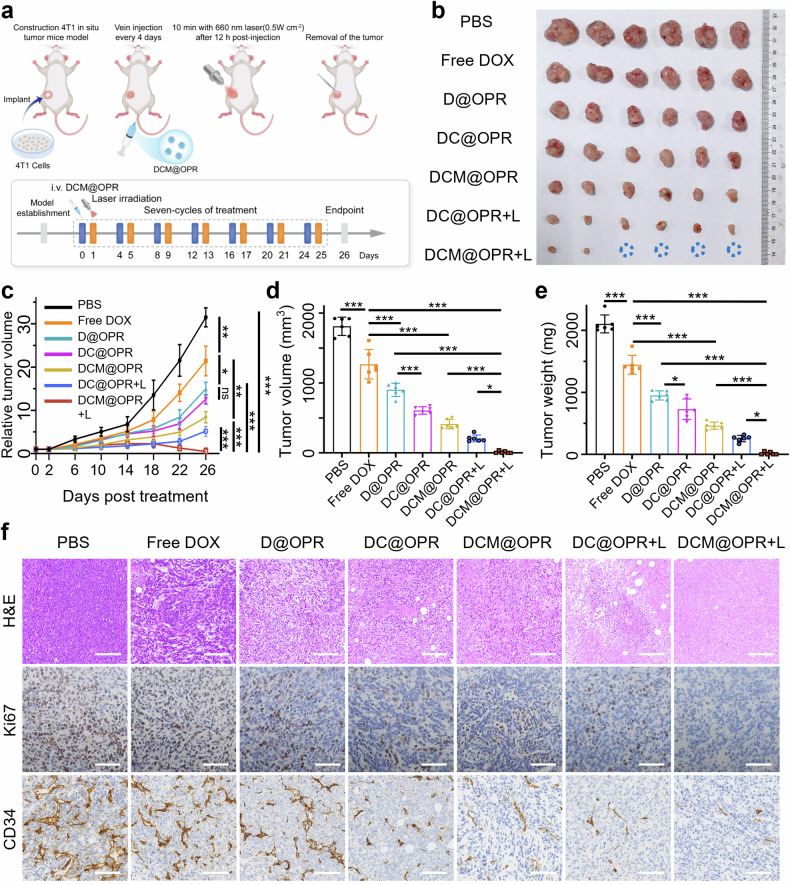
In vivo anti-tumor efficacy of photodynamic gel-bombs (DCM@OPR) in 4T1 in situ tumor-bearing Balb/c mice. **a** Scheme of 4T1 in situ tumor-bearing mice model establishment and treatment procedure. **b** Excised tumors from mice in each group. **c** Relative tumor volumes over time in mice from different treatment groups. *n* = 6 per group. **d** The excised tumor volumes of mice from each group. *n* = 6 per group. **e** The excised tumor weights of mice from each group. *n* = 6 per group. **f** H&E staining of 4T1 tumors from different treatment groups. IHC staining of 4T1 tumors from different treatment groups for cell proliferation using Ki67 and for endothelial vessels using CD34. Scale bars: 100 μm. Data are presented as mean ± SD. *p < 0.05, **p < 0.01, ***p < 0.001, ns means no statistical significance

Next, the anti-tumor efficacy of the photodynamic gel-bombs (DCM@OPR) was evaluated in vivo. In situ tumor-bearing Balb/c mice were constructed using 4T1 cells and subsequently treated with a dosage of 2.5 mg/kg of DOX (1.5 mg/kg of Ce6) administered intravenous injection every 4 days (*n* = 6, Fig. 5a). Seven subgroups were established: PBS, free DOX, D@OPR, DC@OPR, DCM@OPR, DC@OPR + L and DCM@OPR + L. The anti-tumor capacities were determined by measuring tumor volume and tumor weight. The results indicated that the free DOX group exhibited the poorest therapeutic efficacy among all treatment groups, primarily attributed to its rapid metabolism and lack of tumor targeting. The therapeutic efficacy of the DC@OPR groups was found to be superior to that of the D@OPR groups. This difference could be attributed to the cytotoxicity of Ce6 present within the DC@OPR groups (Supplementary Fig. 33). When compared to the DCM@OPR group, the DCM@OPR + L group showed a significantly improved inhibitory effect on tumor growth. Notably, the DCM@OPR + L group exhibited the slowest tumor growth rate, smallest tumor volume, minimum tumor weight, and the strongest inhibitory effect (Fig. 5b–e). We performed flow cytometry detection of ROS in tumor tissues treated with PBS, DCM@OPR, and DCM@OPR + L. We found that the ROS levels were significantly higher in the DCM@OPR + L group compared to the PBS and DCM@OPR groups, indicating that the ROS generated by PDT in the tumor microenvironment played a crucial role in the anti-tumor treatment of DCM@OPR (Supplementary Fig. 34). It is important to note that both the DCM@OPR + L and DC@OPR + L groups demonstrated a similar inhibitory effect during the initial 15 days. In these early stages, tumors could be effectively suppressed by DOX and photodynamically generated ¹O₂ in both groups. However, the tumor microenvironment progressively deteriorated in the later stages of DC@OPR + L groups, which promoted rapid tumor cell proliferation and growth. The presence of MnO_2_ nanoparticles within DCM@OPR enabled the utilization of H_2_O_2_ and H^+^ in the tumor microenvironment to produce O_2_. This process improved the hypoxic microenvironment, thereby enhancing the efficiency of PDT.

To better understand the tumor inhibition mechanisms of the photodynamic gel-bombs (DCM@OPR), we also investigated the synergistic effect between Mn^2+^ and DOX. Recent studies have demonstrated that cGAS/STING can augment the anti-tumor effectiveness of chemotherapeutic drugs.^45^ Furthermore, Mn^2+^ has been shown to promote the activation of cGAS and STING by increasing the production of cGAMP, thereby enhancing the binding affinity of cGAMP/STING.^46^ To confirm the activation of the cGAS/STING pathway, the expression levels of STING and its downstream markers, including IRF3, phosphorylated IRF3 (pIRF3), and IFN-β, were measured by Western blot (WB) in PBS, DC@OPR, CM@OPR, DCM@OPR groups. The results indicated that compared to the DC@OPR (containing DOX, but without Mn^2+^) and CM@OPR (containing Mn^2+^, but without DOX), DCM@OPR (containing DOX and Mn^2+^) could up-regulate the expression of STING, suggesting that the generated Mn^2+^ could synergize with DOX to amplify STING activation. Furthermore, downstream of the cGAS/STING pathway, DCM@OPR was able to increase the expression of pIRF3 and IFN-β (Supplementary Fig. 35). The results suggested that the synergistic action of Mn^2+^ and DOX could ultimately boost the anti-tumor effect by amplifying cGAS/STING activation, which in turn initiate IFN-dependent apoptosis.

To test anti-tumor proliferative capacity, we performed Hematoxylin and Eosin (H&E) staining, immunohistochemistry (IHC), and immunofluorescence (IF) staining on 4T1 tumor tissues after treatment to examine the cell apoptosis, proliferation marker (Ki67) and endothelial lining vascular marker (CD34). These results indicated that the DCM@OPR + L group could promote cell apoptosis. Additionally, this group exhibited significantly lower expression of Ki67 and CD34 compared to other groups, suggesting that the DCM@OPR + L group could effectively suppress tumor growth (Fig. 5f, Supplementary Figs. 36, 37).

Additionally, the biocompatibility and systemic response of photodynamic gel-bombs (DCM@OPR) were further assessed. No significant variations were observed in the body weight of mice across all groups throughout the treatment period (Supplementary Fig. 38). Histological examination using H&E staining revealed no discernible tissue damage in the DCM@OPR + L group (Supplementary Fig. 39). Routine blood tests (including white blood cell (WBC) count, red blood cell (RBC) count, platelet (PLT) count, hemoglobin (HGB), hematocrit (HCT) and neutrophil percentage (NE%)) and serological tests such as creatine kinase (CK), lactate dehydrogenase (LDH), plasma alanine aminotransferase (ALT), aspartate aminotransferase (AST), blood urea nitrogen (BUN) and creatinine (Cre) were measured at 24 h post-injection of DCM@OPR (Supplementary Fig. 40). These results suggested that DCM@OPR could potentially serve as a safe and effective delivery platform for malignant tumor therapy.

Further, to examine the function of c(RGDyk) ligand in vivo, we had also conducted the in vivo anti-tumor efficacy of DCM@OP (without c(RGDyk) ligand) and DCM@OPR (with c(RGDyk) ligand). The results demonstrated that the DCM@OPR + L group exhibited superior therapeutic efficacy compared to the DCM@OP + L group (Supplementary Fig. 41). In addition, we measured the drug concentrations in tumor tissues at different time points. The results revealed that DCM@OPR demonstrated higher tumor accumulation than DCM@OP at the same time intervals (Supplementary Fig. 42). These results indicated that the introduction of c(RGDyk) in DCM@OPR enabled them to enhance the tumor-targeting and accumulation in tumor tissues.

Beyond the in situ 4T1 breast cancer syngraft model, we further investigated the anti-tumor effect of our photodynamic gel-bombs (DCM@OPR) in the in situ MDA-MB-231 breast cancer xenograft model. The in situ MDA-MB-231 breast cancer xenograft model was established by injecting MDA-MB-231 cells into the mammary fat pad of Balb/c nude mice. When palpable tumors could be detected in the abdomen of the mice, indicating the successful establishment of the in situ MDA-MB-231 breast cancer xenograft model. Subsequently, mice were randomly divided into 4 groups (PBS, free DOX, DCM@OPR, DCM@OPR + L) when the average tumor volume reached ~60 mm^3^. These nude mice bearing in situ MDA-MB-231 breast cancer received treatments with different formulations *via* intravenous injection on days 0, 4, 8, 12, 16, and 20. For the DCM@OPR + L group, laser irradiation was applied 12 h after the injection of DCM@OPR. Tumor sizes were periodically measured using calipers to monitor the progression of in situ MDA-MB-231 breast cancer. The results indicated that DCM@OPR significantly inhibited tumor growth compared to the free DOX group. Among them, the DCM@OPR + L group exhibited the slowest tumor growth and well biocompatibility (Supplementary Figs. 43 and 44).

**Fig. 6:**
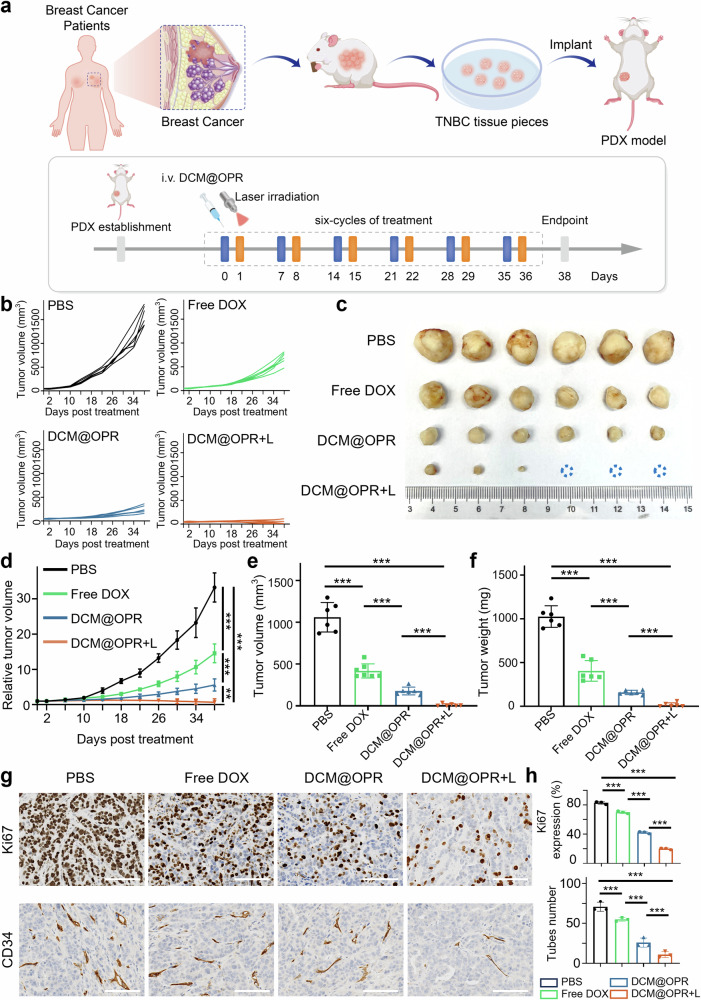
In vivo anti-tumor efficacy of photodynamic gel-bombs (DCM@OPR) in the PDX TNBC models. **a** Scheme of PDX model establishment and treatment procedure. **b** Tumor growth curves of the PDX mouse models. *n* = 6 per group. **c** Image of excised tumors after treatment on day 38. **d** Relative tumor volumes over time in mice from different treatment groups. *n* = 6 per group. **e** Excised tumor volume of different treatment groups. *n* = 6 per group. **f** Excised tumor weight of different treatment groups. *n* = 6 per group. **g** Ki67 and CD34 IHC staining of tumors from mice administered various treatments. *n* = 3 per group. Scale bars: 100 μm. **h** The IHC quantification results in each treatment group. *n* = 3 per group. Data are presented as mean ± SD. *p < 0.05, **p < 0.01, ***p < 0.001

Finally, the anti-tumor growth efficacy and biocompatibility of DCM@OPR were investigated in patient-derived xenograft (PDX) models. As shown in Fig. 6a, fresh tumor tissues from a clinical triple-negative breast cancer (TNBC) patient were subcutaneously transplanted into NOD.Cg-Prkd^cscid^ IL2rgtm^1W*jl*^/SzJ (NSG) mice and these PDX mice were subsequently propagated through mouse-to-mouse transplantation to generate suitable xenograft models. Once the PDX models were successfully established, different formulations were administered via intravenous injection every 7 days. For the DCM@OPR + L group, laser irradiation was applied 12 h post-injection of DCM@OPR. The tumor growth trends of different groups were recorded every 4 days and plotted as growth curves (Fig. 6b, d). After 6 cycles of treatments, free DOX, as a first-line chemotherapeutic agent, showed better tumor growth inhibition than PBS. The DCM@OPR group exhibited superior tumor growth inhibition compared to free DOX, and notably, the DCM@OPR + L group showed the strongest tumor growth inhibition (Fig. 6c, e, f). The Ki67 staining results, along with the CD34 staining results indicated that the DCM@OPR + L group had the lowest cellular proliferation activity and neovascularization (Fig. 6g, h). We also evaluated the biocompatibility of each treatment group in the PDX model, and as shown in Supplementary Fig. 45, there were no significant differences in body weight among all groups throughout the treatment period, and no apparent pathological damage was observed in vital organs (Supplementary Fig. 46).

## Discussion

Over the past decades, nanoparticle-based drug delivery systems have exhibited significant potential in the field of cancer medicine. Several of these nanoparticle-based drug delivery systems, such as albumin-bound Paclitaxel and Doxorubicin hydrochloride liposome, have garnered approval for clinical use owing to their pronounced capacity to diminish the adverse effects associated with these chemotherapeutics while simultaneously augmenting their therapeutic efficacy. However, the effectiveness of these existing systems remains limited by their ability to penetrate deep into solid tumor tissues, due to a series of complex physiological and pathological barriers. To address this issue, previously reported systems have primarily focused on the development of responsive nanocarriers. Their enhanced tumor penetration is attributed to light or other trigger-induced size reduction mechanisms. Nevertheless, their mode of penetration via size reduction still depends on passive diffusion through the dense paracellular matrix, lacking a driving force. Distinctly, our study presents the development of photodynamic gel-bombs (DCM@OPR) that enhance deep tumor penetration efficiency. This is accomplished through a mechanism involving photodynamic-triggered active explosive energy driving and active receptor-mediated transcytosis, resulting in a significant improvement in the therapeutic efficacy of breast cancer treatment.

The photodynamic gel-bombs are fabricated through a simple one-step crosslinking process, which involves mixing Ce6, MnO_2_, and DOX with mPEG-OSA-c(RGDyK) aqueous solution in the presence of Ca^2+^ at room temperature. This process could be easily and efficiently scaled up by controlling the feed concentration of individual components. Different from the previously reported systems, our photodynamic gel-bombs based on Ca^2+^-crosslinked mPEG-alginate hydrogel is not light or other-triggered responsive systems. The blast of the photodynamic gel-bombs is due to the photodynamic-triggered explosive energy. Upon exposure to laser irradiation, these photodynamic gel-bombs could generate a substantial quantity of ROS to provide sufficient explosive energy, affording them to burst into a large number of nanofragments. Subsequently, nanofragments, propelled by the explosive energy (the first driving force), penetrate deep into tumor tissues through the gaps between tumor cells. Meanwhile, when those photodynamic gel-bombs are directly uptaken by tumor cells, they can also fragment into a large number of nanofragments and then promote the escape from lysosomes by photodynamic-triggered explosive energy. In the subsequent stages, these nanofragments can actively penetrate tumor tissues via transcytosis (the secondary driving force). This active process facilitates the transport of drugs across cells, enabling efficient accumulation and profound penetration within tumor tissues. And, we also demonstrate that in the absence of photodynamic-triggered explosive energy, smaller-sized nanofragments are unable to significantly enhance tumor penetration. During the process of photodynamic-triggered treatment, a significant release of therapeutic components (DOX, Ce6, Mn^2+^, Ca^2+^) can be achieved, allowing them highly efficient accumulation into tumor tissues to synergistically exert antitumor effects. The antitumor effects of DOX primarily derive from its interference with the DNA function of tumor cells. Ce6, acting as a photosensitizer, generates cytotoxic ROS under laser irradiation, leading to tumor cell death. The MnO_2_ nanoparticles contained within the photodynamic gel-bombs can leverage H_2_O_2_ and H^+^ in the tumor microenvironment to produce O_2_. This process alleviates hypoxia and fosters the generation of ROS, thereby enhancing the efficiency of photodynamic therapy. Concurrently, the resultant Mn^2+^ synergistically combines with DOX to co-stimulate the cGAS/STING pathway, potentially conferring anti-tumor properties. The substantial release of Ca^2+^ during the explosion further induces tumor cell apoptosis. This antitumor effectiveness is demonstrated in three distinct animal models: the 4T1 syngraft model, the MDA-MB-231 xenograft model, and the PDX model. Therefore, the photodynamic gel-bombs significantly enhance treatment efficacy for tumors through excellent synergetic effects among these therapeutic components.

Although the photodynamic gel bombs demonstrate more profound results in the treatment of solid tumors by enhancing their deep permeation capacity, concerns remain regarding their potential for treating deep-seated tumors due to limited light penetration in future clinical applications. In clinical, PDT has demonstrated considerable potential in treating a variety of subcutaneous and cutaneous tumors, including but not limited to skin cancer, breast cancer, brain tumors, esophageal cancer, and bladder cancer. Accordingly, the photosensitizers commonly used in clinical practice include 5-Aminolevulinic Acid (5-ALA, 635 nm), Hematoporphyrin Derivative (HpD, 630 nm), Temoporfin (652 nm), among others. To address issues, in future applications aimed at treating deep-seated tumors, there is potential to replace current light sources and photosensitizers, such as employing light sources and photosensitizers in near-infrared region II making them suitable for photodynamic therapy in deep tissue. For example, light sources in the near-infrared region II can penetrate ~20 mm into the skin.^47^ Photosensitizers of near-infrared region II, such as IR1064 (1064 nm) exhibit superior photosensitizing properties.^48^ Moreover, while photodynamic gel-bombs have shown notable therapeutic potential in numerous animal models, their translation to clinical practice remains questionable due to the absence of human clinical trial data. Comprehensive investigations are urgently needed to elucidate their pharmacokinetic profiles, systemic toxicity, and long-term therapeutic outcomes. Therefore, future research should prioritize the development of innovative clinical application platforms to promote the application of these promising synergistic combination therapy strategies, which is crucial for enhancing tumor penetration and improving treatment efficacy against solid tumors.

Collectively, the photodynamic gel-bombs demonstrated exceptional deep penetration capabilities via photodynamic-triggered explosive energy propulsion and receptor-mediated transcytosis. This mechanism, based on active propulsion, stands in contrast to previously described responsive systems that rely on size reduction-mediated passive diffusion. As a result, the photodynamic gel-bombs may offer enhanced efficiency in deep penetration and subsequent tumor treatment, showcasing significant promise for clinical applications in targeted therapy. Meanwhile, in the realm of targeted therapy for clinical applications, the integration of multi-strategy synergy by introducing various therapeutic components into one drug delivery system remains a promising avenue of future research because it can significantly enhance the treatment efficiency for solid tumors.

In summary, we have developed photodynamic gel-bombs, enabling them to penetrate deeply into tumor tissues, significantly enhancing the therapeutic efficacy of breast cancer. The photodynamic gel-bombs facilitate enhanced deep penetration through photodynamic-triggered explosive energy driving and receptor-mediated transcytosis. This mechanism differs from previously reported responsive systems that rely on size reduction-mediated passive diffusion. We believe that the photodynamic gel-bombs facilitate profound penetration into tumor tissues for efficient therapeutic component delivery and downstream synergistic therapies, offering a promising avenue for the treatment of solid tumors.

## Materials and methods

### Preparation of oxidized sodium alginate (OSA)

Sodium alginate, renowned for its superior biocompatibility, non-toxicity, plentiful carboxyl groups facilitating easy modification, and commendable degradability, is extensively utilized in diverse fields such as drug carriers, wound dressings, food additives, and cosmetic moisturizers.^49^ Consequently, we selected it as our preferred drug carrier. The OSA was synthesized as follows: 3.00 g of sodium alginate (ALG, S817374, Macklin, Shanghai) was dissolved in 280 mL of deionized water at room temperature. Simultaneously, 2.95 g of sodium periodate (NaIO_4_, S817518, Macklin, Shanghai) was dissolved in 20 mL of deionized water. Under subdued light conditions, the NaIO_4_ solution was added dropwise to the ALG aqueous solution. After stirring for 12 h, the resulting product was slowly added to 400 mL of cold anhydrous ethanol and allowed to precipitate overnight at 4 °C. Subsequently, the precipitate was centrifuged and washed three times with ethanol. The washed precipitate was dissolved in deionized water and freeze-dried to obtain the powdered OSA.

### Preparation of OSA-mPEG (OP)

PEGylated drug carriers can avoid rapid clearance by the reticuloendothelial system (RES), thereby reducing immune responses and extending their circulation time in the bloodstream.^50^ The OP was synthesized as follows: 0.5 g of OSA was dissolved in 25.00 mL of pH = 5.5 PBS solution. Subsequently, N-hydroxysuccinimide (NHS, 20 mg) and 1-ethyl-3-(3-dimethylaminopropyl) carbodiimide (EDC, 20 mg) were added, and the reaction proceeded for 30 min. Next, 0.30 g of Methoxy PEG Amine (mPEG-NH_2_, PS1-N-2K) powder was dissolved in 5.00 mL of pH = 5.5 PBS solution and added dropwise to the OSA solution described above, allowing the reaction to proceed 24 h. Unreacted OSA was then removed by ethanol precipitation, while the resulting OSA-mPEG was dissolved in ethanol. The resultant product was dialyzed using a dialysis membrane (MWCO 14000 Da) in deionized water for 48 h to remove unreacted mPEG-NH_2_. The dialyzed product was freeze-dried to obtain OP. Characterization of OP using FTIR spectra (Agilent, Cary 630).

### Preparation of mPEG-OSA-c(RGDyK) (OPR)

The OPR was synthesized as follows: NHS (20 mg) and EDC (20 mg) were added to 5.00 mg/mL of OP aqueous solution, and the reaction proceeded for 30 min. c(RGDyk) (RP50011, BAM BIOTECH CO., LTD) (20 mg) was dissolved in deionized water and added dropwise to the above solution, ensuring a mass ratio of OP: c(RGDyK) = 50:6. The reaction was stirred for 12 h, resulting in the formation of mPEG-OSA-c(RGDyK) (OPR). The resulting product was dialyzed using a dialysis membrane (MWCO 14,000 Da) in deionized water for 48 h to remove unreacted c(RGDyK) and the possible side product. The FTIR spectra and ^1^H NMR spectra were used to confirm the chemical compositions of OPR.

### Synthesis of the photodynamic gel-bombs (DCM@OPR)

OPR (5 mg, pH = 8), 150 µL of 0.20 M calcium chloride (CaCl_2_, C805228, Macklin, Shanghai) solution, 60.00 mg polyvinyl alcohol (PVA, 767382, Macklin, Shanghai), 1 mg Doxorubicin hydrochloride (DOX, D807083, Macklin, Shanghai), 1 mg Ce6 (C302679, Shanghai Yuanye Bio-Technology Co., Ltd), and 120 µL of 600 µg/mL MnO_2_ nanoparticles (JK-09-006, njjikebiotec Co., Ltd) were mixed with deionized water to a total volume of 3 mL and stirred for 12 h at 800 rpm. The resulting reaction mixture was washed twice with deionized water by centrifugation at 10,000 rpm for 10 min to remove substances that did not participate in the reaction. The final product was resuspended with deionized water to obtain the photodynamic gel-bombs (DCM@OPR) for further experiments.

### Synthesis of the Cy5 labeled photodynamic gel-bombs (Cy5-DCM@OPR)

The typical synthesis of Cy5-DCM@OPR involved adding 10 mg of DCM@OPR, 5 mg of EDC, and 5 mg of NHS into 1.5 mL of deionized water. This mixture was then left to react at room temperature for 12 h. Following this reaction, the resulting product, Cy5-DCM@OPR, was subjected to dialysis in deionized water using a dialysis membrane with a molecular weight cut-off (MWCO) of 3500 Da for 48 h. This process was undertaken to remove any unreacted Cy5. The final product was then lyophilized.

### Characterization of the photodynamic gel-bombs (DCM@OPR) and DCM@OPR nanofragments

TEM (JEOL-1400Flash, Japan) and CLSM (Nikon, Japan) were used to observe the morphology of DCM@OPR before being exposed to laser irradiation and after being exposed to laser irradiation (660 nm, 30 mW/cm^2^) for 1, 5, and 10 min. CLSM was also used to observe the localization of DOX and Ce6 within DCM@OPR. DLS (Malvern Instruments, UK) was employed to measure the size distribution and Zeta potential of the photodynamic gel-bombs. UV–Vis spectrophotometer was used to study the absorption spectra of DOX, Ce6 and DCM@OPR in the 300-700 nm range. Elemental manganese in DCM@OPR was detected using a scanning electron microscope energy spectrum analysis system (SEM-EDX, EDAX Octane Elect Plus, USA). The concentrations of DOX and Ce6 in solution were detected using a UV–Vis spectrophotometer, and the concentrations of elemental Mn^2+^ and Ca^2+^ were quantified using inductively ICP-MS (PerkinElmer, USA). The drug encapsulation efficiency (EE) and loading efficiency (LE) were calculated using the following equation:

$${\rm{EE}}\,( \% )=({\rm{weight}}\; {\rm{of}}\; {\rm{loaded}}\; {\rm{drug}}/{\rm{total}}\; {\rm{weight}}\; {\rm{of}}\; {\rm{drug}}\; {\rm{input}})\times 100 \%$$

$${\rm{LE}}\,( \% )=({\rm{weight}}\; {\rm{of}}\; {\rm{loaded}}\; {\rm{drug}}/{\rm{total}}\; {\rm{weight}}\; {\rm{of}}\; {\rm{particles}})\times 100 \% .$$

In addition, Gel Permeation Chromatography (GPC) was used to detect the molecular weight of different particles, and FTIR spectra and ^1^H NMR spectra were used to detect the molecular structural features of different particles.

### Detection of ROS in vitro

To detect the generation of ROS, a DPBF probe was used as an indicator. In brief, 10 μL 20 μM DPBF was mixed with 2 mL photodynamic gel-bombs (30 μg/mL of Ce6 equivalent). Then, the absorbance of DPBF at 417 nm was recorded by a UV–visible spectrophotometer after being irradiated by laser (660 nm, 30 mW/cm^2^) for different time. DPBF solution served as the control.

### Drug release in vitro

The supernatant was collected by centrifugation (10,000 rpm, 10 min) before or after the DCM@OPR was exposed to laser irradiation (660 nm, 30 mW/cm^2^, 10 min). The concentration of DOX and Ce6 was then detected by UV–vis spectrophotometer, while the content of Mn^2+^ and Ca^2+^ was determined by ICP-MS.

### Stability evaluation of photodynamic gel-bombs (DCM@OPR)

DCM@OPR was placed in 10% FBS and stored at room temperature. The particle size and PDI were monitored by DLS over a period of 168 h.

### Calculation of the complexation ratio (*n*) and the complexation constant (*K*) between Ca^2+^ and alginate

The volume of *V*_1_, the concentration of *C*_1_ ALG solution was added to the prepared volume of V_2_ concentration of *C*_2_ excess CaCl_2_ solution. Following this, sufficient cross-linking was conducted. The concentration of Ca^2+^ in the supernatant was then quantified using ICP-MS, yielding a calcium concentration (*C*_3_). The precipitated complex underwent a series of purification steps: it was first rinsed five times with deionized water and subsequently dried and weighed to obtain a mass (*m*). Following this, the precipitate was introduced into the volume of *V* of deionized water (*V* = *V*_1_ + *V*_2_) and allowed to be set aside for 12 h. The concentration of Ca^2+^ in the resulting supernatant was then quantified using ICP-MS, yielding a measured value of *C*_4_.

The *n* of alginate to Ca^2+^ in calcium alginate:

5 $$n\,=\,\frac{m-\left({C}_{2}{V}_{2}-{C}_{3}V\right){M}_{2}}{\left({C}_{2}{V}_{2}-{C}_{3}V\right){M}_{1}}$$

*K* between alginate and Ca^2+^:

6 $$K\,=\,\frac{{C}_{1}}{{C}_{2}\,\times \,{{C}_{3}}^{n}}=\frac{\left({C}_{2}{V}_{2}-{C}_{3}V\right)}{V{n}^{n}{{C}_{4}}^{n+1}}$$

where *M*_1_ represented the molecular weight of alginate: 175.112 g/mol and *M*_2_ represented the molecular weight of Ca^2+^: 40.08 g/mol. The units for m and V were mg and mL, respectively. *C*_1_, *C*_2_, and *C*_3_ represent the concentrations of calcium alginate, Ca^2+^, and alginate ions formed after reaching complexation equilibrium, respectively, where *n* was the complexation ratio of alginate to Ca^2+^ in calcium alginate. Through rigorous experimentation and calculation, the *n* and *K* between Ca^2+^ and alginate were ascertained to be 2.16 and 2.97 × 10^5^.

### Drug release profile of nanofragments

The release profile of DOX from the nanofragments was investigated in 0.1 M acetate buffer (pH 6.5). Specifically, the nanofragments were dissolved in the acetate buffer and enclosed in a dialysis membrane with a dialysis membrane (MWCO 3500) and then immersed in 100 mL of acetate buffer by gently shaking at 37 °C. At designated time intervals, aliquots of the dialysate were collected for analysis using a UV–visible spectrophotometer, after which an equal volume of fresh buffer was added to maintain a constant volume. DOX concentrations were determined by a UV–vis spectrometer (480 nm) according to standard curves at the corresponding buffer solutions. The group was measured in triplicate.

### Cell culture

The murine breast cancer cells 4T1 and human breast cancer cells MDA-MB-231 were obtained from the Cell Bank of Type Culture Collection of the Chinese Academy of Sciences (Shanghai, China). 4T1 cells or MDA-MB-231 cells were cultured in 1640 medium or DMEM medium supplemented with 10% (v/v) FBS (Keygen BioTECH) and 1% (v/v) penicillin-streptomycin, and routinely incubated at 37 °C with 5% CO_2_ in a humidified environment. CELLSAVING (C40100, New Cell & Molecular Biotech) was used for cell storage.

### Ability to penetrate multicellular 4T1 spheroids

To assess the deep penetration capability in tumors, multicellular 4T1 spheroids were generated. Briefly, 3 × 10^3^ cells/well were seeded in each well of the 96-well plates (PCP011096, PROMETHE) coated with agarose and incubated for 168 h to form multicellular 4T1 spheroids. Then, the tumor spheroids were meticulously aspirated and carefully placed in confocal dishes (NEST Biotechnology). The tumor spheroids were respectively treated with free DOX, free Ce6, free Cy5-OPR, D@OP, and DCM@OPR (DOX equivalent: 2.5 μg/mL in free DOX group, D@OP and DCM@OPR group, Ce6 equivalent: 2.5 μg/mL in free Ce6 group, Cy5 equivalent: 2.5 μg/mL in free Cy5-OPR group) for 4 h. After co-incubation, the tumor spheroids were washed with PBS, fixed in 4% paraformaldehyde for 15 min, and then assessed for penetration capability using CLSM.

### Ability to penetrate heterospheroids

Heterospheroids were generated using 4T1 cells and cancer-associated fibroblasts (CAFs) to assess the deep penetration capability. Briefly, 1.5 × 10^3^ 4T1 cells and 1.5 × 10^3^ CAFs were mixed and seeded in each well of the 96-well plates coated with agarose and incubated for 168 h to form heterospheroids. Then, the heterospheroids were meticulously aspirated and carefully placed in confocal dishes. The heterospheroids were respectively treated with DCM@OPR or DCM@OPR nanofragments (DOX equivalent: 2.5 μg/mL) for 4 h. For the DCM@OPR + L group, laser irradiation (660 nm, 30 mW/cm^2^, 10 min) was administered after 2 h exposure to DCM@OPR. Then heterospheroids were incubated with CD44 antibody (60224-1, Proteintech) and FAP antibody (84018-4-RR, Proteintech) overnight at 4 °C. After incubation with secondary antibodies, the heterospheroids were washed with PBS, fixed in 4% paraformaldehyde for 15 min, and then assessed for penetration capability using CLSM.

### Cellular uptake of DCM@OPR nanofragment

Flow cytometry was employed to ascertain the cellular uptake of DCM@OPR nanofragment. 4T1 cells were cultured in 6-well plates at a density of 3 × 10^5^ cells/well and incubated at 37 °C for 12 h. Control groups, free DOX groups, DCM@OPR nanofragment groups, and DCM@OPR nanofragments + Laser (DCM@OPR nanofragments+L) groups (DOX equivalent: 2.5 μg/mL) were established. The DCM@OPR nanofragments + L groups underwent laser irradiation (660 nm, 30 mW/cm^2^, 10 min) after 2 h co-incubation with cells and DCM@OPR. Following 4 h co-incubation, all groups were washed twice with PBS, centrifuged, and resuspended in 200 μL PBS. Flow cytometry (Becton Dickinson, USA) was utilized to detect DOX (PE-A channel). The results were analyzed using FlowJo 10.8 software.

### Endocytosis pathway detection of DCM@OPR nanofragments

DCM@OPR nanofragments were obtained after 10 min of laser irradiation (660 nm, 30 mW/cm^2^, 10 min) on DCM@OPR. 4T1 cells were then seeded in 6-well plates at a concentration of 3 × 10^5^ cells/well and incubated at 37 °C for 12 h. Subsequently, pharmacological inhibitors such as chlorpromazine (CPZ) (10 μg/mL), methyl-β-cyclodextrin (MβCD) (2.5 mM), and amiloride (15.1 µg/mL) were introduced to the cells and incubated for 1 h at 37 °C. Following this, DCM@OPR nanofragments were added and further incubated for 2 h at 37 °C. To investigate the influence of temperature on the endocytosis of DCM@OPR nanofragments, cells were pre-incubated at 4 °C for 1 h before the addition of DCM@OPR nanofragments, which were then incubated for another 2 h at 4 °C. The cells were subsequently washed with PBS solution, centrifuged, and resuspended in PBS. Flow cytometry was employed to detect the PE-A signal, and inhibition rates were calculated as a percentage of that internalized in the control group.

### Transcytosis ability of DCM@OPR nanofragments

A method previously described in the literature.^51–53^ Firstly, 4T1 cells were seeded in 6-well plates (3 × 10^5^ cells in 1 mL medium per well) and incubated for 12 h at 37 °C, which were designated as A cells. Subsequently, free DOX and DCM@OPR nanofragments (DOX concentration: 2.5 µg/mL) were added to the A cells in the wells and incubated for 4 h at 37 °C. After that, the drug-containing medium of A cells was washed with a sterile PBS solution three times. The same number of Hoechst 33342-labeled 4T1 cells (designated as B cells) were then added to the A cells in each well and incubated at a constant temperature for 1.5 h, forming A + B cells. Cells were subsequently trypsinized, washed with cold PBS three times, and re-suspended in 500 μL PBS. The DOX signal was detected by flow cytometry. The autofluorescence of untreated cells was used as the control. Data analysis was performed using FlowJo 10.8 software. All experiments were repeated three times, and the results shown here are representative of three independent experiments.

### Detection of intracellular ROS levels

4T1 cells were seeded in 12-well plates at a density of 1.5 × 10^5^ cells/well and incubated at 37 °C for 12 h. Following this, they were treated with either DC@OPR or DCM@OPR (DOX equivalent: 2.5 μg/mL, Ce6 equivalent: 1.5 μg/mL) for an additional 12 h under hypoxic conditions (oxygen partial pressure of 1%). Subsequently, a 2′,7′-Dichlorodihydrofluorescein diacetate (DCFH-DA) probe (ROS assay kits, S0033S, Beyotime Biotechnology Co Ltd.) at a concentration of 10 μM was added to each well and exposed to laser irradiation (660 nm, 30 mW/cm^2^, 10 min) or not. The cells were further incubated at 37 °C for 20 min and washed three times with serum-free 1640 medium. The intracellular levels of ROS were subsequently assessed using flow cytometry (FITC channel) or CLSM.

### Colocalization study of CM@OPR and lysosomes

CM@OPR group and CM@OPR with laser irradiation (CM@OPR + L) group were set up. 4T1 cells were seeded in 12-well plates at a density of 2 × 10^4^ cells/well and incubated at 37 °C for 12 h and then co-incubated with CM@OPR (Ce6 equivalent: 1.5 μg/mL) for 4 h. For the CM@OPR + L group, laser irradiation (660 nm, 30 mW/cm^2^, 10 min) was applied after the cells were co-incubated with CM@OPR for 4 h. The medium was replaced with fresh serum-free culture medium preheated to 37 °C containing LysoTracker Red DND-99 (300 nM) (C1046, YEASEN Biotech Co., Ltd.)and further incubated for 1.5 h. Then cells were washed with PBS, fixed with 4% paraformaldehyde for 15 min, stained with Hoechst 33342 (C1028, Beyotime Biotechnology Co. Ltd.) for 5 min, and finally washed with PBS. CLSM was used to observe the distribution of fluorescence within the cells.

### Acridine orange (AO) staining

4T1 cells were seeded in confocal dishes at a density of 1 × 10^5^ cells/well and incubated at 37 °C for 12 h and then co-incubated with CM@OPR (Ce6 equivalent: 1.5 μg/mL) for 4 h. For the CM@OPR + L group, laser irradiation (660 nm, 30 mW/cm^2^, 10 min) was applied after the cells were co-incubated with CM@OPR for 4 h. Then cells were stained with 1 μM AO solution (MedChemExpress, HY-101879) in culture medium at 37 °C for 20 min. Then cells were washed with PBS, stained with Hoechst 33342 for 5 min, and finally washed with PBS. CLSM was used to observe the distribution of fluorescence within the cells.

### Intracellular Internalization

Intracellular internalization was observed using a CLSM. 4T1 cells were seeded at a concentration of 3 × 10^4^ cells/well on glass coverslips in 12-well plates and incubated at 37 °C for 12 h. The free DOX groups, the DCM@OPR groups, and the DCM@OPR + L groups were set up (DOX equivalent: 2.5 μg/mL). Among them, the DCM@OPR + L groups were given laser irradiation (660 nm, 30 mW/cm^2^, 10 min) after co-incubation of cells with DCM@OPR for 2 h. After 4 h of different treatments, the cells were washed with PBS; fixed with 4% paraformaldehyde for 15 min, followed by staining with Hoechst 33342 for 5 min, and finally washed with PBS. Fluorescence distribution within the cells was observed using a CLSM.

### Intracellular drug accumulation

Flow cytometry was used to detect intracellular accumulation of DOX and Ce6. 4T1 cells were seeded in 6-well plates at a concentration of 3 × 10^5^ cells/well and incubated at 37 °C for 12 h. Control groups, free DOX groups, DCM@OPR groups, and DCM@OPR + L groups (DOX equivalent: 2.5 μg/mL, Ce6 equivalent: 1.5 μg/mL) were established. In DCM@OPR + L groups, laser irradiation (660 nm, 30 mW/cm^2^, 10 min) was applied after 2 h of co-incubation of cells with the DCM@OPR. After 4 h of co-incubation, all groups were washed twice with PBS, centrifuged, and resuspended in 200 μL PBS. Flow cytometry was used to detect DOX (PE-A channel) and Ce6 (FITC channel). The results were analyzed by FlowJo 10.8 software.

ICP-MS was used to detect intracellular accumulation of Mn^2+^ and Ca^2+^. 4T1 cells were seeded in 6-well plates at a concentration of 3 × 10^5^ cells/well and incubated at 37 °C for 12 h. The PBS groups, DCM@OPR groups, and DCM@OPR + L groups were set up (DOX equivalent: 2.5 μg/mL, Ce6 equivalent: 1.5 μg/mL). Among them, the DCM@OPR + L groups were given laser irradiation (660 nm, 30 mW/cm^2^, 10 min) after co-incubation of cells with DCM@OPR for 2 h. After 4 h of co-incubation, the cells were washed twice with PBS and mixed with concentrated nitric acid overnight, then microwave digestion and filtered through a 0.22 μm microporous membrane, and the concentrations of Mn^2+^ and Ca^2+^ were detected by ICP-MS.

### In vitro cytotoxicity assay

To determine the inhibitory effect on the proliferation of 4T1 tumor cells, cell viability was assessed using the MTT assay kit (M8180, Beijing Solarbio Science & Technology Co., Ltd.). Groups of control, free DOX, D@OPR, DC@OPR, DCM@OPR, DC@OPR + L and DCM@OPR + L were set up. 4T1 cells were cultured in 96-well plates at a density of 3.5 × 10^3^ cells/well. After 12 h of incubation to allow cell adhesion, free DOX, D@OPR, DC@OPR, and DCM@OPR (DOX equivalent: 2.5 μg/mL, Ce6 equivalent: 1.5 μg/mL) were added to each well and incubated at 37 °C for 24 h. At 12 h of incubation, laser irradiation (660 nm, 30 mW/cm^2^, 10 min) treatment was given or not. Cell activity was calculated by measuring absorbance values at 490 nm using a microplate reader (Synergy2, Bio-Tek, USA).

In the same way, free DOX, DCM@OPR, and DCM@OPR + L groups were set up to determine the inhibitory effect on the proliferation of MDA-MB-231 tumor cells. In addition, the cytotoxicity of different concentrations of Ce6@OPR and free OPR was detected using the MTT assay.

### Calculation of the CI

To quantitatively analyze the drug synergy of photodynamic gel-bombs, the Chou–Talalay method^54,55^ was employed to calculate the CI. The CI can quantitatively assess the nature (synergistic, additive, antagonistic) and extent of drug interactions when different drugs are used in combination.

The CI is defined as follows:

$${{\rm {CI}}}=\frac{{{D}}_{1}}{{{D}}_{{X}1}}+\frac{{{D}}_{2}}{{{D}}_{{X}2}}$$

where *D*_1_ and *D*_2_ represent the doses of drug 1 and drug 2 required to achieve a certain level of drug effect (e.g., 50% inhibition of cell viability) when used in combination. *D*_*x*1_ and *D*_*x*2_ represent the doses of each single drug required to achieve the same level of drug effect when used individually. The CI value must correspond to the respective level of drug effect to be meaningful. Specifically, CI < 1 indicates synergism, CI = 1 indicates an additive effect, CI > 1 indicates antagonism.

### Animal and animal tumor model

Female Balb/c nude mice and Balb/c mice, weighing 18–20 g at 6–8 weeks, were purchased from the Experimental Animal Center of Southern Medical University. The mice were housed under specific pathogen-free (SPF) conditions. To establish 4T1 in situ breast cancer syngraft model or MDA-MB-231 in situ breast cancer xenograft model, anesthesia was induced by intraperitoneal injection of 1% pentobarbital sodium (8 μL/g body weight). Anesthetized animals were placed in the prone position and fixed on the operating table. An ~1 cm long skin incision was made along the midline of the abdomen, and 4T1 cells (1 × 10^6^) or MDA-MB-231 (1 × 10^7^) suspended in 25 μL of PBS were injected into the mammary fat pad of female Balb/c mice or Balb/c nude mice. After confirming the absence of leakage, the skin was sutured.

PDX models were further established. The PDX model used in this study was established and provided by the Department of Breast Surgery, Zhujiang Hospital, Southern Medical University. As described previously,^56^ the tumor tissues were obtained with written informed consent from the patient, and all procedures were conducted in accordance with ethical standards. PDX models were established in immunodeficiency mice. Once established, PDXs were expanded in six-week-old NSG mice to obtain the third-generation (P3) for experiments. When the maximum diameter of the PDXs’ tumor reached approximately 5 mm, the mice were allocated to various treatment groups.

All operations were trained, and the experimental protocol was reviewed and approved by the Ethics Committee of Zhujiang Hospital, Southern Medical University (Approval No. LAEC-2024-310).

### In vivo biodistribution and tumor-targeting capacities of the photodynamic gel-bombs (DCM@OPR)

In situ 4T1 tumor-bearing nude mice models were constructed as described above. To study the distribution and tumor-targeting capacities of DCM@OPR, we modified the particles with Cy7. The typical synthesis consisted of adding 10 mg of DCM@OPR, 50 μg of sulfo-Cy7 amine (R-H-7109, Xi’an Ruixi Biological Technology Co., Ltd), 5 mg of EDC and 5 mg of NHS to 1.5 mL of deionized water, and react at room temperature for 12 h. The unreacted Sulfo-Cy7 amino acid was subsequently removed by dialysis in deionized water for 48 h using a dialysis membrane with a molecular weight cut-off (MWCO) of 3500 Da. Free Cy7, Cy7-DCM@OP, and Cy7-DCM@OPR (at a concentration of 0.05 mg/kg Cy7) were injected through the tail vein into 4T1 in situ tumor-bearing Balb/c nude mice. Cy7 fluorescence was detected at 3, 6, 12, 24, 48, and 72 h post-injection using an in vivo imaging system (IVIS Lumina II, Caliper, USA). At 24, 48, and 72 h post-injection, mice were euthanized, and major organs, as well as the tumors, were excised. The relative accumulation of Cy7 fluorescence was measured using the IVIS.

### In vivo deep tissue penetration capacity

To study the deep tissue penetration capacity of DCM@OPR in 4T1 cancer, Panoramic Scan (3DHISTECH CaseViewer, Hungary) was used to observe the fluorescence intensity of DOX and Ce6 in different groups. Different experimental groups were included with the free DOX group, DCM@OPR group and DCM@OPR + L group, respectively. 4T1 in situ tumor-bearing Balb/c mice were injected via the tail vein with free DOX and DCM@OPR (DOX equivalent: 2.5 mg/kg, Ce6 equivalent: 1.5 mg/kg). For the DCM@OPR + L group, laser irradiation (660 nm, 500 mW/cm^2^, 10 min) was administered 12 h after the injection of DCM@OPR. At 24 h post-injection, the mice were euthanized, and the tumors were collected and fixed in 4% paraformaldehyde for 24 h, dehydrated in a 30% sucrose solution, embedded in OCT compound, sectioned, and stained with Hoechst 33342. Finally, the fluorescence intensity of the tissue sections was observed using a Panoramic Scan.

To assess the ability of DCM@OPR to accumulate within the tumor microenvironment and undergo deep penetration, 4T1 tumor-bearing Balb/c mice were given a dose of 2.5 mg/kg of DOX (1.5 mg/kg of Ce6) each substance via tail vein injection. The mice were anesthetized and transcardially perfused at various times. For the laser irradiation group, laser irradiation (660 nm, 500 mW/cm^2^, 10 min) was administered 12 h post-drug administration. And, then, the breast cancers were excised carefully and then fixed in 4% paraformaldehyde for 24 h and further dehydrated in graded ethanol. After sectioning, the samples were stained with Hoechst 33342 and CD34 antibodies (15H1, HUABIO) to assess the distance of the drug from blood vessels. F4/80 antibodies (4G4, HUABIO)and CD44 antibodies (JE64-01, HUABIO)were used to mark macrophages and tumor cells, respectively, to detect the accumulation of the drug in the tumor microenvironment.

### Accumulation of drugs in major organs and tumor tissues in vivo

To determine the tumor-targeting capacity of DCM@OPR, DOX, Ce6, Mn^2+^ and Ca^2+^ in tumor tissues of different subgroups were semi-quantified/quantified. The experimental subgroups consisted of free DOX/free Ce6/PBS, DCM@OPR, and DCM@OPR + L. Free DOX/free Ce6/PBS and DCM@OPR (DOX equivalent: 2.5 mg/kg, Ce6 equivalent: 1.5 mg/kg) were injected via tail vein into 4T1 in situ tumor-bearing Balb/c mice respectively. For the DCM@OPR + L group, laser irradiation (660 nm, 500 mW/cm^2^, 10 min) was applied 12 h after injection. All groups of mice were euthanized 24 h after injection, and tumor tissues, as well as major organs (heart, liver, spleen, lungs, kidneys), were collected. To semi-quantify the delivery efficiency of DOX and Ce6, all tissues were washed, dried, weighed, and deproteinized with acetonitrile, followed by grinding and centrifugation at 12,000 rpm for 10 min. Aspirate the supernatant and the concentration of DOX and Ce6 were quantified using a cell imaging multi-mode reader (BioTek Cytation5, USA). To quantify the delivery efficiency of Mn^2+^ and Ca^2+^, all tissues were washed, dried, weighed, and immersed in concentrated nitric acid overnight, processed using a microwave ablator, then filtered through a 0.22 μm filter membrane, and the concentrations of Mn^2+^ and Ca^2+^ were determined using ICP-MS.

### In vivo analysis of the effect of photodynamic gel bombs (DCM@OPR) on tumor cells and tumor tissues before and after laser irradiation using TEM

TEM was utilized to observe the effect of photodynamic gel bombs (DCM@OPR) on 4T1 breast tumor cells and tumor tissues in vivo before and after laser irradiation. In brief, two groups were established: the DCM@OPR group and the DCM@OPR + L group. DCM@OPR (DOX equivalent: 2.5 mg/kg, Ce6 equivalent: 1.5 mg/kg) was injected into 4T1 in situ tumor-bearing Balb/c mice via the tail vein. After 12 h injection, laser irradiation (660 nm, 500 mW/cm^2^, 10 min) was administered or not. After 24 h post-injection, the mice were anesthetized, and the tumor tissue was carefully collected and fixed in 2.5% glutaraldehyde. Subsequently, tumor tissues were sent to the Central Laboratory of Southern Medical University for the preparation of the TEM samples and finally, using TEM for section observation and elemental analysis.

### Anti-tumor efficacy in vivo

When the average tumor volume of 4T1 in situ tumor-bearing Balb/c mice reached approximately 60 mm^3^, the mice were randomly divided into 7 groups: PBS group, free DOX group, D@OPR group, DC@OPR group, DCM@OPR group, DC@OPR + L group and DCM@OPR + L group (6 mice/group). Injections were administered every 4 days for a total of 7 injections via the tail vein, with a DOX equivalent of 2.5 mg/kg and Ce6 equivalent of 1.5 mg/kg. For the DC@OPR + L group and DCM@OPR + L group, laser irradiation (660 nm, 500 mW/cm^2^, 10 min) was administered 12 h after injection. Tumor volumes were monitored by measuring the vertical diameter using calipers. Volumes in vivo were estimated using the formula: *V* = *L* × *W*^2^ × 1/2 and volume ex vivo were calculated by the formula: *V* = *π*/6 × *L* × *W* × *H* (*V*, tumor volume; *L*, tumor length; *W*, tumor width; *H*, tumor height). Starting from the 2nd day of treatment, tumor volumes, and mice body weights were monitored every 4 days. Mice were euthanized on the 26th day after the first treatment. Heart, liver, spleen, lung, kidney, and tumor of mice from each group were collected and stained by H&E staining kits (DH0006, Leagene). As described in the previous study,^57,58^ IHC and IF analyses were performed by labeling proliferation marker (Ki67, HA721115, HUABIO) and endothelial-lined blood vessel marker (CD34, ET1606-11, HUABIO) antibodies. Thereafter, a second-antibody kit (abs996, absin) was used at room temperature. The H&E and IHC sections were observed with an optical microscope (Nikon, Japan) and IF sections were observed by CLSM.

The anti-tumor efficacy of DCM@OP and DCM@OPR were evaluated in 4T1 tumor-bearing Balb/c mice. PBS group, DCM@OP + L group, and DCM@OPR + L group were established. Injections were administered via tail vein every 4 days for a total of 5 injections at 2.5 mg/kg DOX equivalent (1.5 mg/kg Ce6 equivalent). Laser irradiation (660 nm, 500 mW/cm^2^, 10 min) was performed 12 h after injection. Tumor volume and mice body weight were monitored every 4 days starting from the 2nd day of treatment. Mice were euthanized on day 22 after the first treatment. Tumors were collected from each group of mice and further analyzed by IHC.

Expand beyond 4T1 cell line to test efficacy in other solid tumor, we constructed MDA-MB-231 in situ breast cancer xenograft model to evaluated gel-bombs DCM@OPR for broader coverage. Similarly, when the average tumor volume reached approximately 60 mm^3^, different treatments were administered to the corresponding groups, and tumors were subsequently collected at day 22 after 6-cycles treatment. Biocompatibility and anti-tumor efficacy were evaluated by similar methods as 4T1 in situ tumor-bearing Balb/c mice.

Due to the difference between cell-derived xenograft (CDX) models and actual patient tumors, TNBC PDX models were established on female NSG mice. Once PDX models were successfully established, different formulations (DOX equivalent of 2.5 mg/kg, Ce6 equivalent of 1.5 mg/kg) were administered via intravenous injection every 7 days. Laser irradiation (660 nm, 500 mw/cm^2^, 10 min) was administered 12 h after injection for the DCM@OPR + L group. Starting from the 2nd day of treatment, tumor volumes, and mice body weights were monitored every 4 days. After 38 days of treatment, anti-tumor growth efficacy and biocompatibility were evaluated. Labeling proliferation marker (Ki67, bsm-60738R, Bioss USA) and endothelial-lined blood vessel marker (CD34, bs-8996R, Bioss USA) antibodies were used for IHC analyses.

### In vivo ROS levels in tumor microenvironment

PBS, DCM@OPR, and DCM@OPR + L groups were established on 4T1 in situ tumor-bearing Balb/c mice to evaluate ROS levels in tumor microenvironment. PBS and DCM@OPR (DOX equivalent of 2.5 mg/kg; Ce6 equivalent of 1.5 mg/kg) were injected via the tail vein. At 24 h post-injection, laser irradiation (660 nm, 500 mW/cm^2^, 10 min) was administered to DCM@OPR + L group. Then tumors of each group were collected. As previously reported,^59^ tumor tissues were mechanically minced and subsequently digested enzymatically with collagenase and dispase. Tumor cells in the resulting single-cell suspension were labeled with Live/Dead Fixable Blue (L34961, Thermo Fisher, USA) and ROS Fluorometric Assay Kit (E-BC-K138-F, Elabscience® Biotechnology Co., Ltd.). Flow cytometry was utilized and the results were analyzed using FlowJo 10.8 software.

### WB analysis

The expression of STING, IRF3, pIRF3, and IFN-β were detected by WB. PBS, DC@OPR, CM@OPR, DCM@OPR groups were established on 4T1 in situ tumor-bearing Balb/c mice. DC@OPR (DOX equivalent of 2.5 mg/kg), CM@OPR (Mn^2+^ equivalent of 0.1 mg/kg), DCM@OPR (DOX equivalent of 2.5 mg/kg, Mn^2+^ equivalent of 0.1 mg/kg) were injected via the tail vein (6 mice/group). Then tumors of each group were collected. Proteins were extracted from tumor tissues of different groups and separated by 12% sodium dodecyl sulfate–polyacrylamide gel electrophoresis (SDS-PAGE). Subsequently, the proteins were incubated on polyvinylidene difluoride (PVDF) membranes with STING (1:1000, A21051, ABclonal, China), IRF3 (1:1000, A11118, ABclonal, China), p-IRF3 (1:1000, AP0995, ABclonal, China), IFN-β (1:1000, A25818, ABclonal, China), and GAPDH (1:1000, A19056, ABclonal, China) at 4 °C overnight. The signals were observed with SuperSignal West Pico PLUS (Thermo Fisher, USA) and analyzed using ImageLab software.

### In vivo biocompatibility evaluation

To assess the biocompatibility and systemic response of DCM@OPR, we investigated the effects of different treatments on blood biochemical indices and routine blood indicators using healthy Balb/c mice. Healthy Balb/c mice were divided into PBS group, free DOX group, D@OPR group, DC@OPR group, DCM@OPR group, DC@OPR + L group, and DCM@OPR + L group (3 mice/group). Injections via the tail vein with a DOX equivalent of 2.5 mg/kg and Ce6 equivalent of 1.5 mg/kg. For the DC@OPR + L group and DCM@OPR + L group, laser irradiation (660 nm, 500 mw/cm^2^, 10 min) was administered 12 h after injection. Blood was collected through the eye sockets of mice 24 h after injection. Routine blood tests (including WBC count, RBC count, HGB, PLT count, HCT, and NE% were measured to assess bone marrow toxicity. Serological biochemical indices such as CK, LDH, ALT, AST, BUN, and Cre were analyzed to assess cardiac toxicity, liver toxicity, and kidney toxicity. ALT Elisa Kit (ELK1921) and AST Elisa Kit (ELK1778), were purchased from ELK Biotechnology. BUN Elisa Kit (JL-T1013), and Cre assay kit (JL-T0929) were purchased from Jianglai Biology, Shanghai.

### Tumor targeting ability

4T1 in situ tumor-bearing Balb/c mice were divided into two groups and administered DCM@OP and DCM@OPR intravenously at doses of 2.5 mg/kg DOX to evaluate their tumor-targeting ability in vivo. The tumors were collected at different time points (0.083, 0.5, 3, 6, 12, 24, 48, 72 h) after injection, and then were washed, dried, weighed, and deproteinized with acetonitrile, followed by grinding and centrifugation at 12,000 rpm for 10 min. Aspirate the supernatant and quantify the concentration of DOX in the supernatant using a cell imaging multi-mode reader (BioTek Cytation5, USA).

### Statistical analysis

All data are expressed as mean ± standard deviation (SD). Statistical significance was analyzed using unpaired Student’s *t*-test when comparing 2 independent groups, one-way ANOVA with Bonferroni correction when comparing three or more independent groups, and two-way ANOVA with repeated measures when analyzing two nominal predictor variables (independent variables) on a continuous outcome variable (dependent variable). Statistical significance was expressed as *p < 0.05, **p < 0.01, ***p < 0.001.

## Supplementary information

Supplementary materials

## Data Availability

The data that support the findings of this study are available from the corresponding author upon reasonable request.
