# Supplementary material for: Photodynamic gel-bombs enhance tumor penetration and downstream synergistic therapies
Source: Signal Transduct Target Ther. 2025 Mar 19;10:94. doi: 10.1038/s41392-025-02186-y (PMC11920195; doi:10.1038/s41392-025-02186-y)
Supplement: Supplementary file 1 — Supplementary materials [file 41392_2025_2186_MOESM1_ESM.docx]

Supplementary Materials for

Photodynamic Gel-Bombs Enhance Tumor Penetration and Downstream Synergistic Therapies

Xiaole Bai^1,2,#^, Fanliang Meng^3,#^, Xuejiao Wang^2^, Linyun He^2,4^, Chao Fan^2,5^, Liangjie Tian^1^, Yangning Zhang^2^, Jiahao Pan^2^, Qun Wu^2^, Xiangrong Hao^2^, Ying Wang^2^, Bo-Feng Zhu^1,6*^, Jun-Bing Fan^2*^ and Bin Cong^1,7*^ .

Correspondences to: Bin Cong ([cong6406@hebmu.edu.cn](mailto:cong6406@hebmu.edu.cn));

Jun-Bing Fan ([fjb2012@mail.ipc.ac.cn](mailto:fjb2012@mail.ipc.ac.cn));

Bo-Feng Zhu (zhubofeng7372@126.com).

**This PDF file includes:**

Figures. S1 to S46

Tables S1 to S6


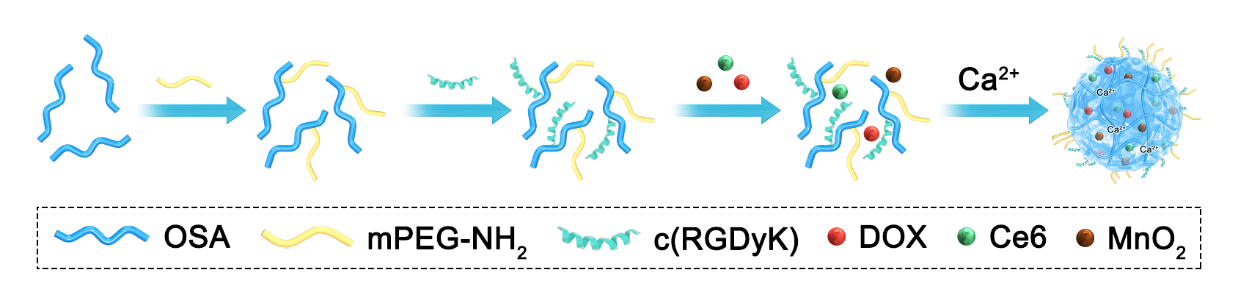


## Figure. S1. Schematic of fabrication of photodynamic gel-bombs (DCM@OPR).


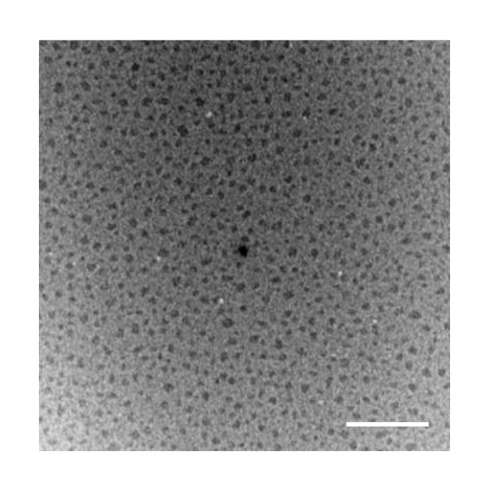


## Figure. S2. The morphology of MnO_2_ nanoparticles. Scale bar: 50 nm.


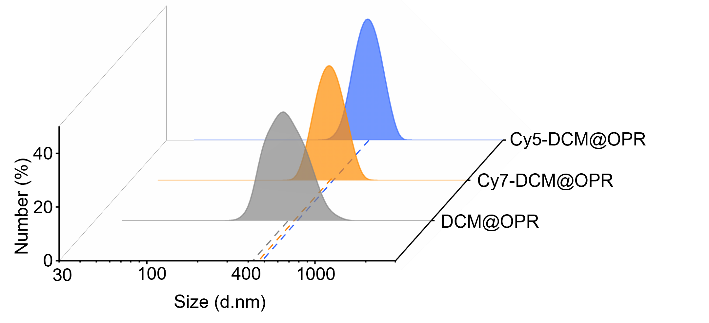


**Figure. S3.** The size distribution of DCM@OPR, Cy5-DCM@OPR and Cy7-DCM@OPR detected by DLS.


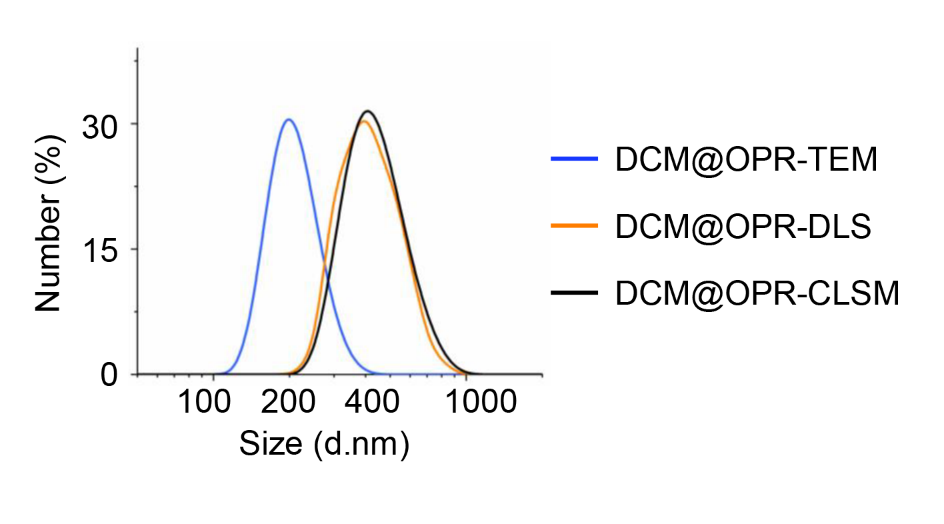


## Figure. S4. The sizes of the photodynamic gel-bombs (DCM@OPR) in swelling and shrinking states. In TEM images, the sizes of DCM@OPR were approximately shrunk in half after dehydration. The size of the photodynamic gel-bombs (DCM@OPR) from TEM and CLSM was measured by image J ver. 1.54m software. And use GraphPad Prism ver. 10.1.2 software to create particle size distribution curves based on the measured diameter data.


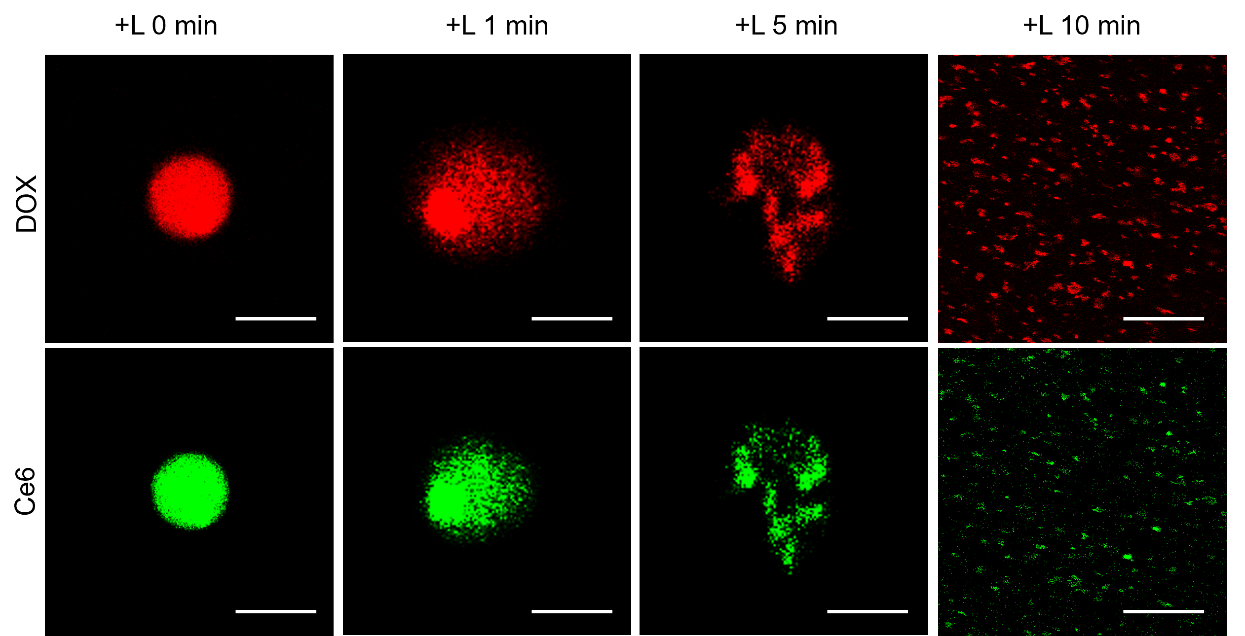


**Figure. S5.** The distribution of DOX and Ce6 within the photodynamic gel-bombs (DCM@OPR). Upon exposure to laser irradiation, the photodynamic gel-bombs exhibited bomb-like properties, which gradually expanded and then burst into a large number of nanofragments accompanying the extension of laser irradiation time. Red and green fluorescence represented DOX and Ce6, respectively. Scale bars: 400 nm.


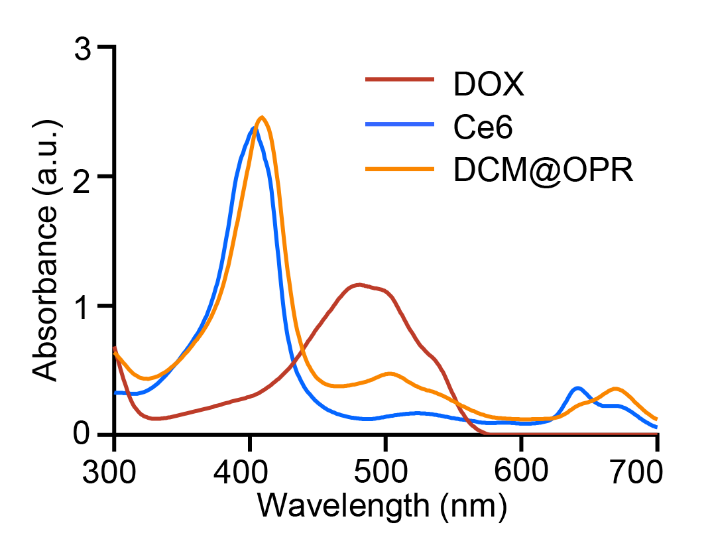


## Figure. S6. UV-Vis spectra of DOX, Ce6 and photodynamic gel-bombs (DCM@OPR) in water.


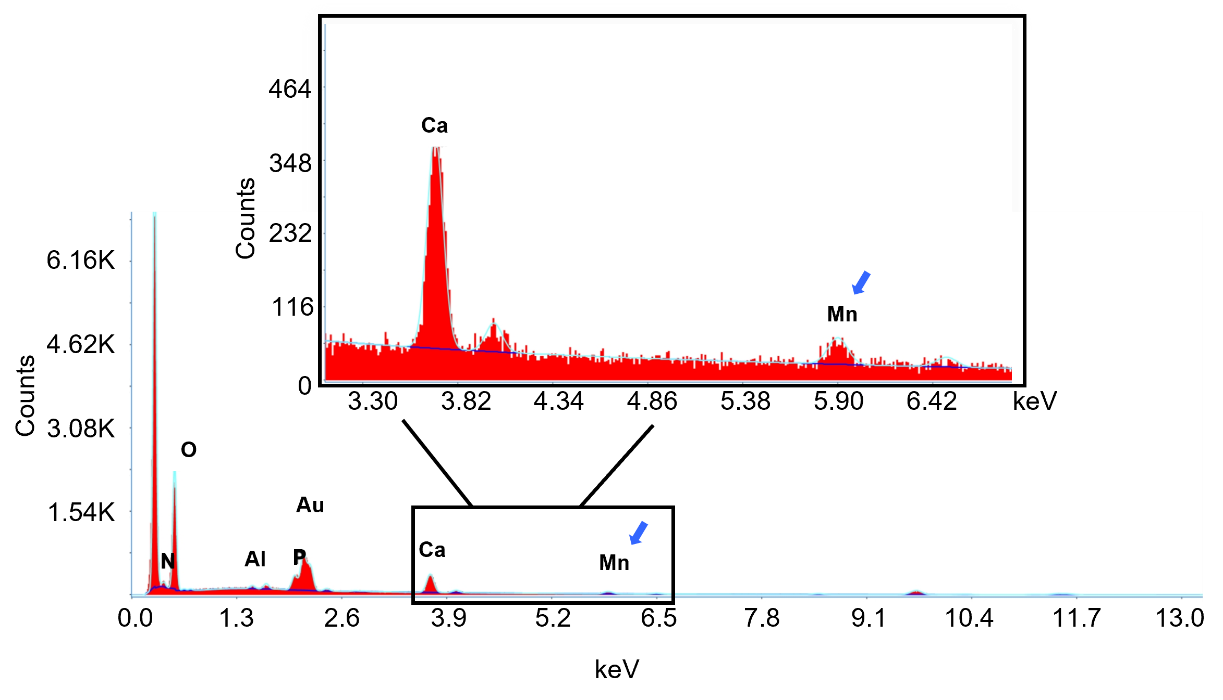


**Figure. S7.** Scanning electron microscope energy-dispersive X-ray spectroscopy (SEM-EDS) analysis of photodynamic gel-bombs (DCM@OPR).


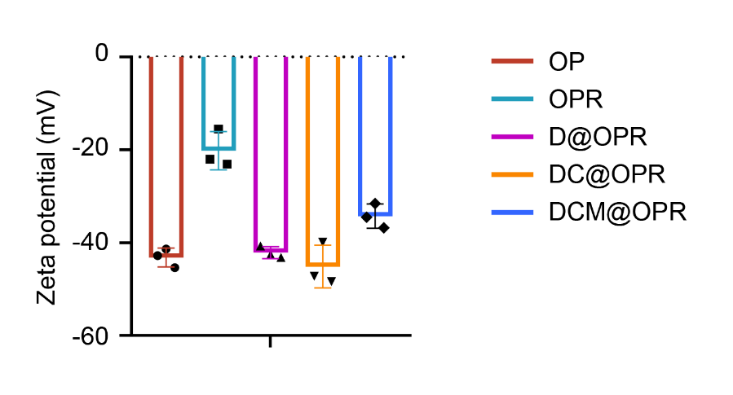


## Figure. S8. The zeta potentials of OP, OPR, D@OPR, DC@OPR, DCM@OPR using DLS. Data are presented as mean ± SD (n = 3).


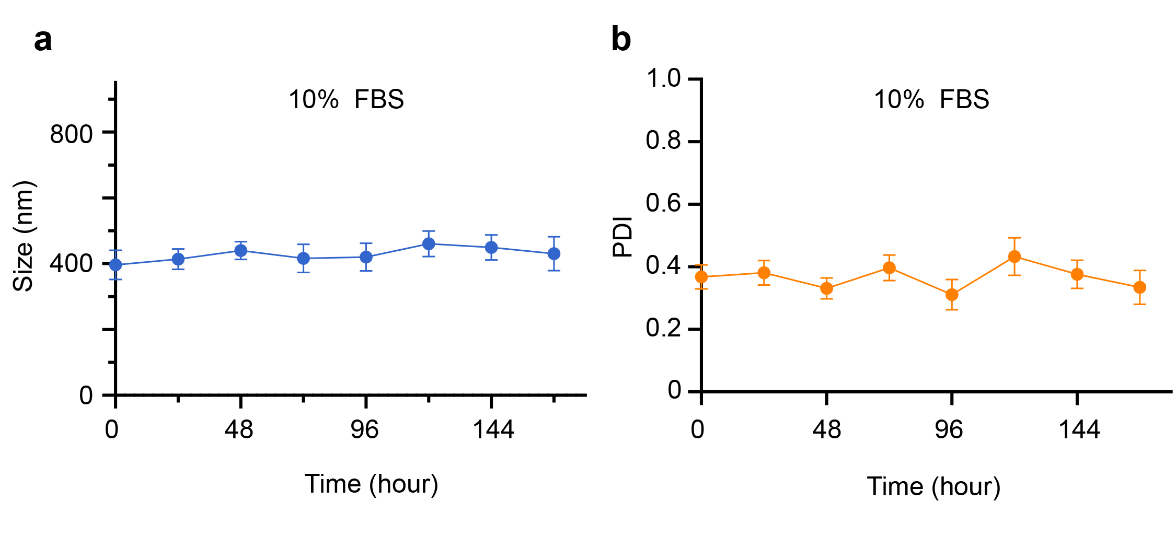


**Figure. S9.** The stability of photodynamic gel-bombs (DCM@OPR). Structural stability of DCM@OPR in 10% FBS after 168 h of storage at room temperature. **a**) The sizes and **b**) PDI of DCM@OPR. Data are presented as mean ± SD (n = 3).


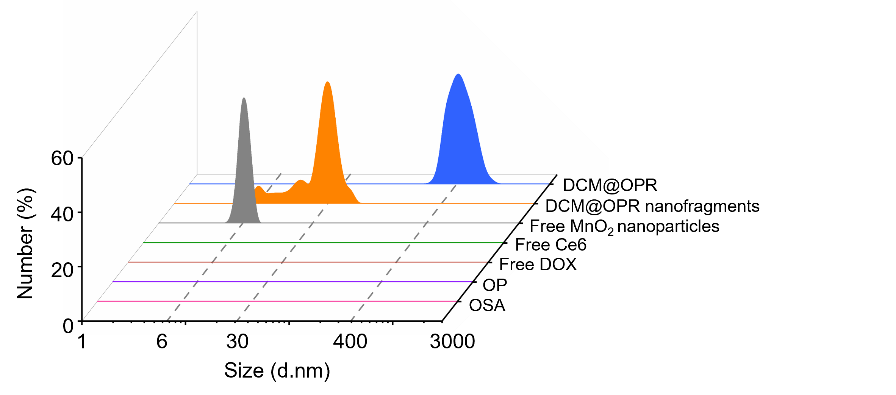


**Figure. S10.** The size distribution of OSA, OSA-mPEG (OP), free DOX, free Ce6, free MnO_2_ nanoparticles, DCM@OPR nanofragments and DCM@OPR gel-bombs detected by DLS.


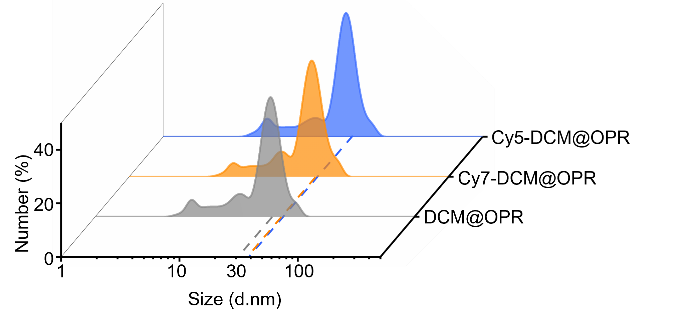


**Figure. S11.** The size distribution of DCM@OPR nanofragments, Cy5-DCM@OPR nanofragments and Cy7-DCM@OPR nanofragments detected by DLS.


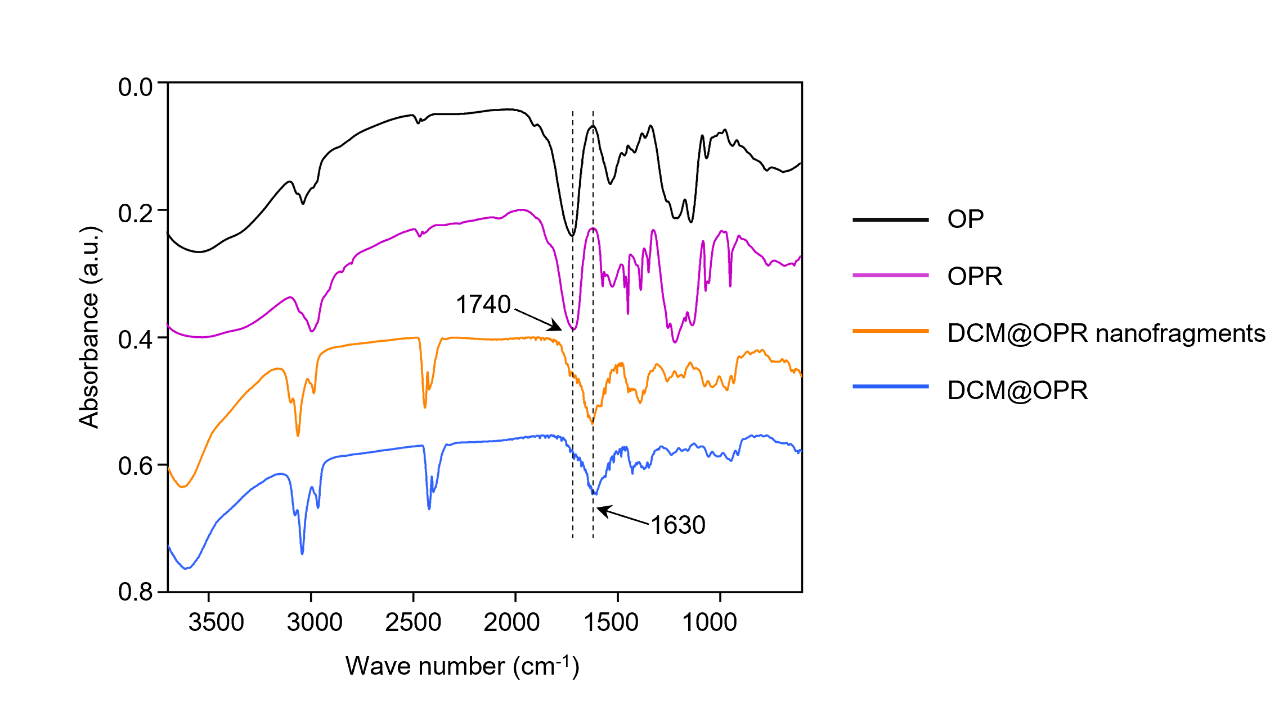


**Figure. S12.** FTIR spectra of OP, OPR, DCM@OPR nanofragments and DCM@OPR. FTIR results indicate the forming of –CONH as the absorbance of the C=O stretching vibration at 1740 cm^-1^ in OP and OPR (*Langmuir.* **2018**. *34*: 416-424; *J. Mater. Chem. B.* **2017**. *5*: 2840-2848). While, a significant Schiff base bond is proven by the remarkable absorbance band at 1630 cm^-1^ corresponding to the symmetric stretching vibration of C=N in DCM@OPR, which is also in accordance with previously reported works (*Bioact. Mater*. **2023**. *25*: 273-290).


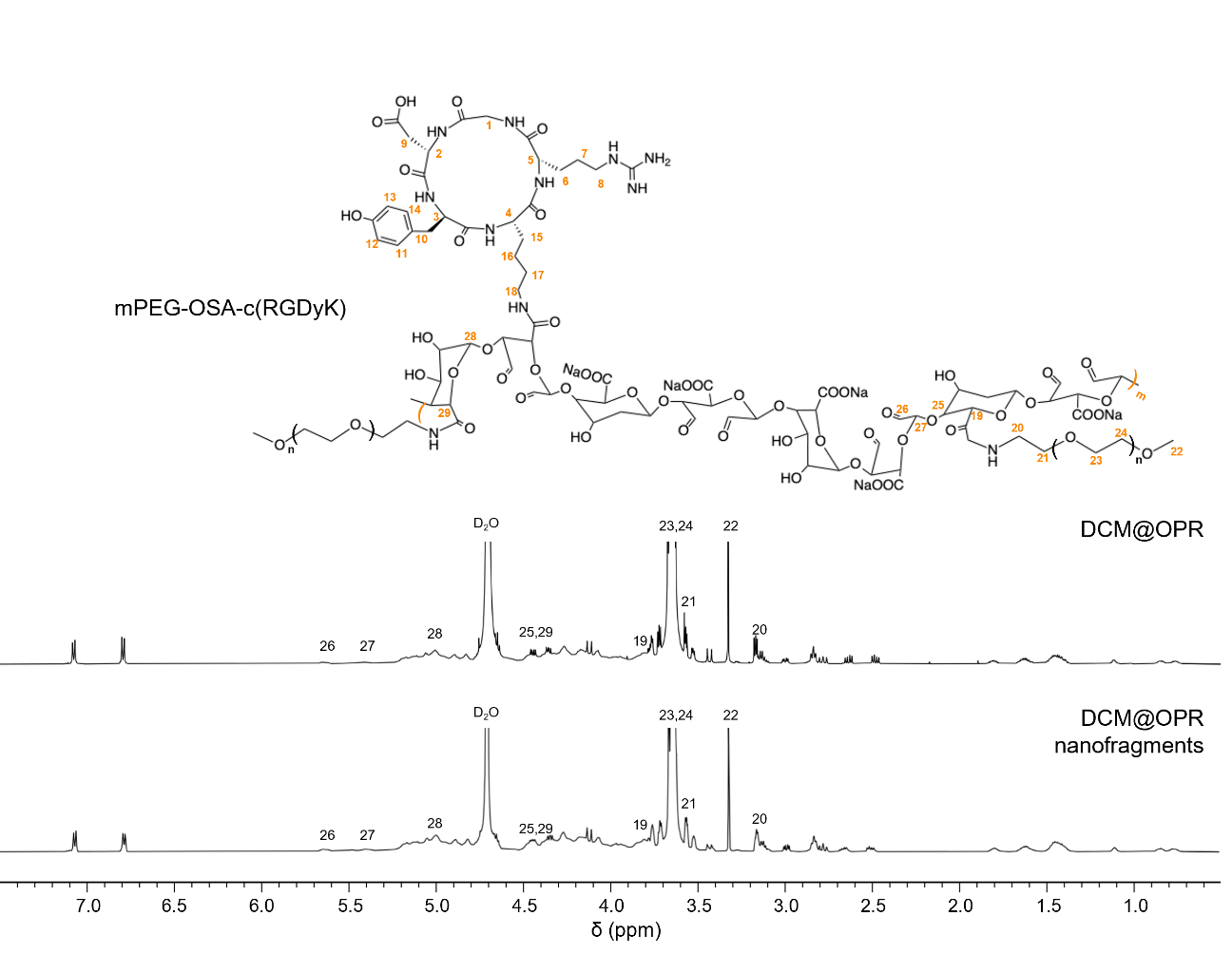


**Figure. S13.** ^1^H NMR spectra of DCM@OPR and DCM@OPR nanofragments in D_2_O.


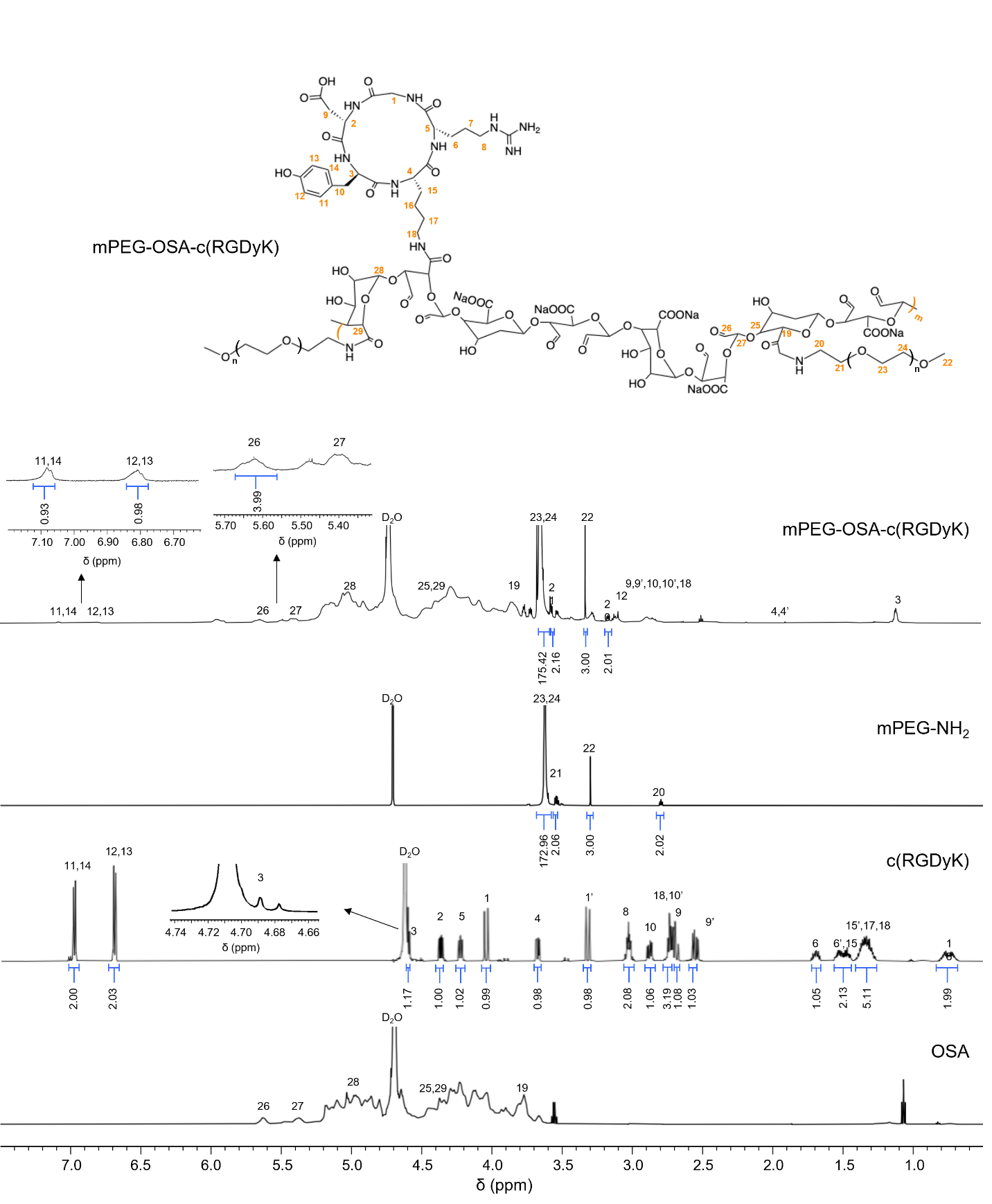


**Figure. S14.** ^1^H NMR of OSA, c(RGDyK), mPEG-NH_2_, and mPEG-OSA-c(RGDyK) in D_2_O. According to the ^1^H NMR, the degree of substitution of mPEG-NH_2_ and c(RGDyK) on the alginate was calculated to be 0.25 and 0.125, respectively. In detail, the integral values at positions 12 and 13 for c(RGDyK) has 1 H with an integral value of 0.98, which is served as a reference. At 26, alginate has 1 H with integral value of 3.99 and at 20, mPEG-NH_2_ has 2 Hs with integral value of 2.01. So the ratio of alginate: mPEG-NH_2_:c(RGDyK) is 8 : 2 : 1 from the integral areas. The degree of substitution of mPEG-NH_2_ and c(RGDyK) on alginate is 0.25 (2/8) and 0.125 (1/8), respectively.


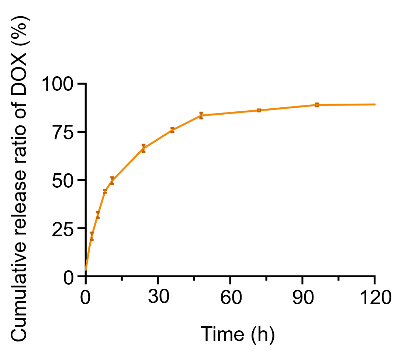


**Figure. S15.** The cumulative release of DOX from nanofragments at pH 6.5. The data were shown as mean ± SD (n = 3).


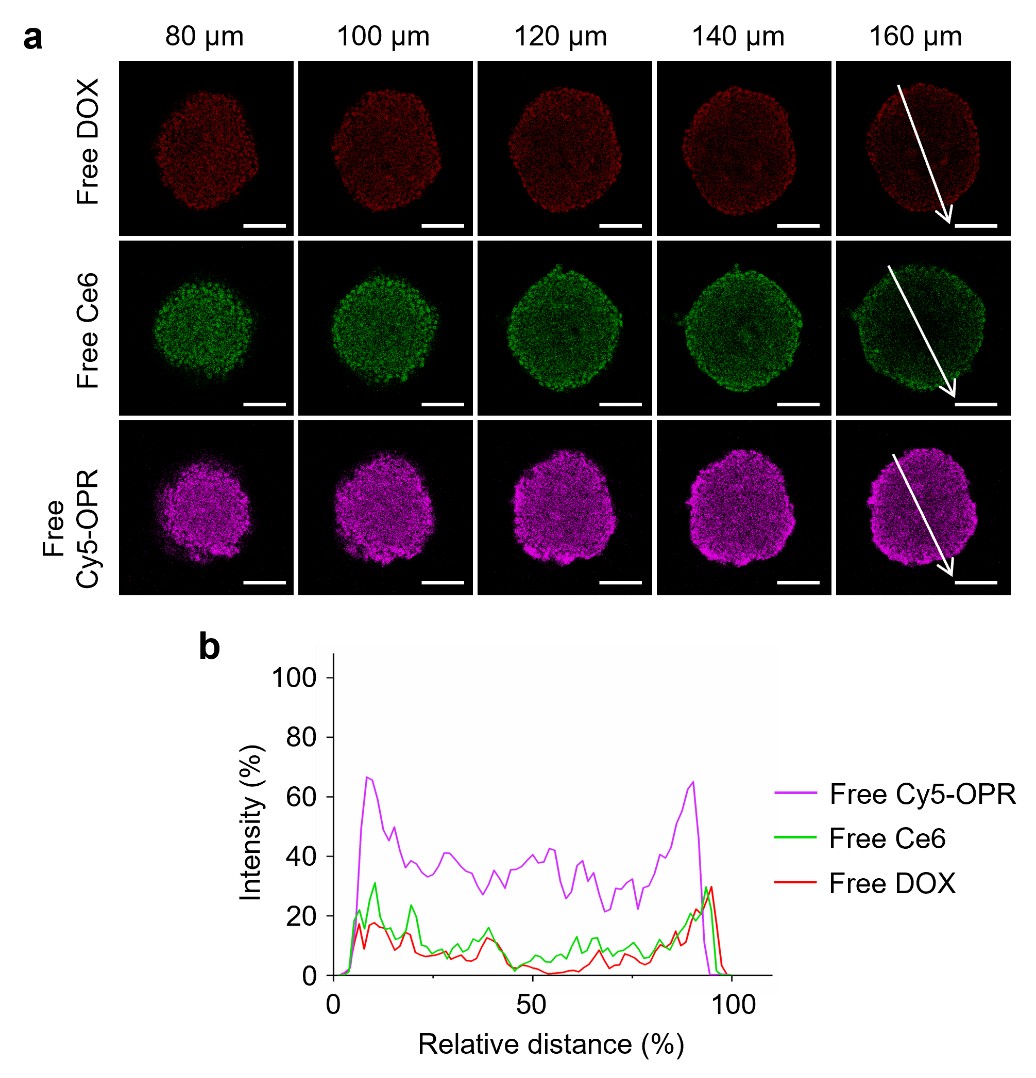


**Figure. S16.** **a**) The penetration ability of free DOX, free Ce6 and free Cy5-OPR (with same amount of DOX, Ce6 and Cy5) in 4T1 multicellular tumor spheroids. Scale bars: 100 μm. **b**) Corresponding fluorescence intensity along the white line at 160 μm scanning depth in 4T1 multicellular tumor spheroids.


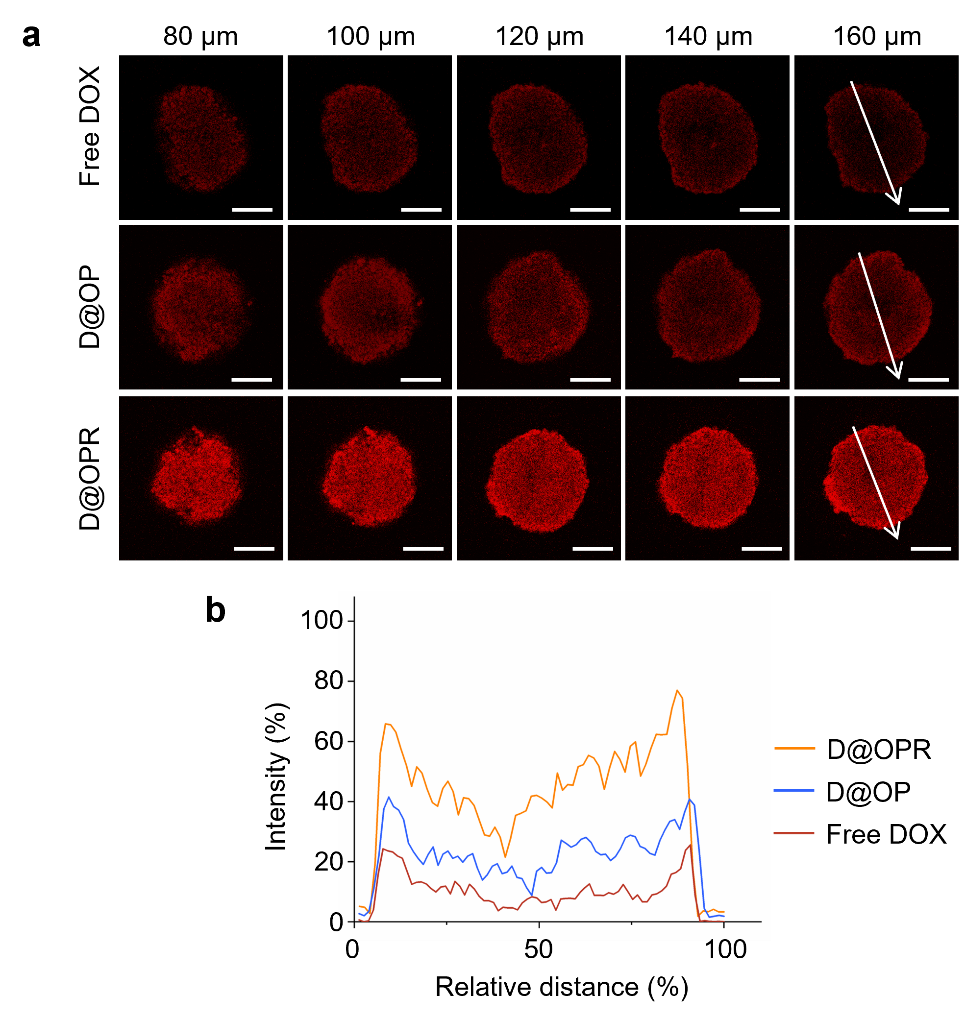


**Figure. S17. a**) The penetration ability of free DOX, D@OP and D@OPR in 4T1 multicellular tumor spheroids. Scale bars: 100 μm. **b**) Corresponding fluorescence intensity along the white line at 160 μm scanning depth in 4T1 multicellular tumor spheroids.


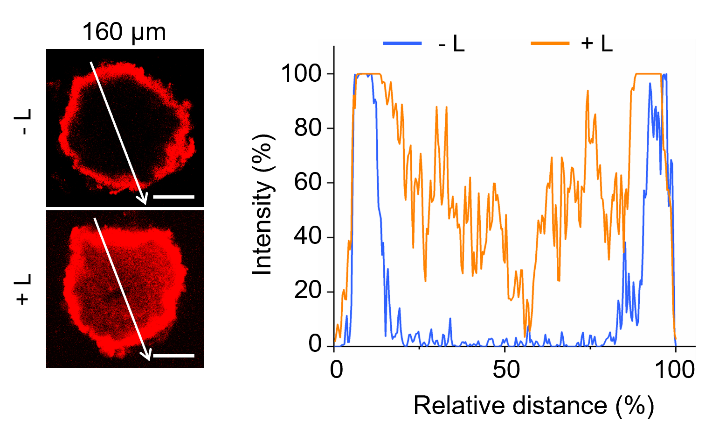


**Figure. S18.** Corresponding fluorescence intensity along the white line at 160 μm scanning depth in heterospheroids of DCM@OPR before and after laser irradiation. Scale bars: 100 μm.


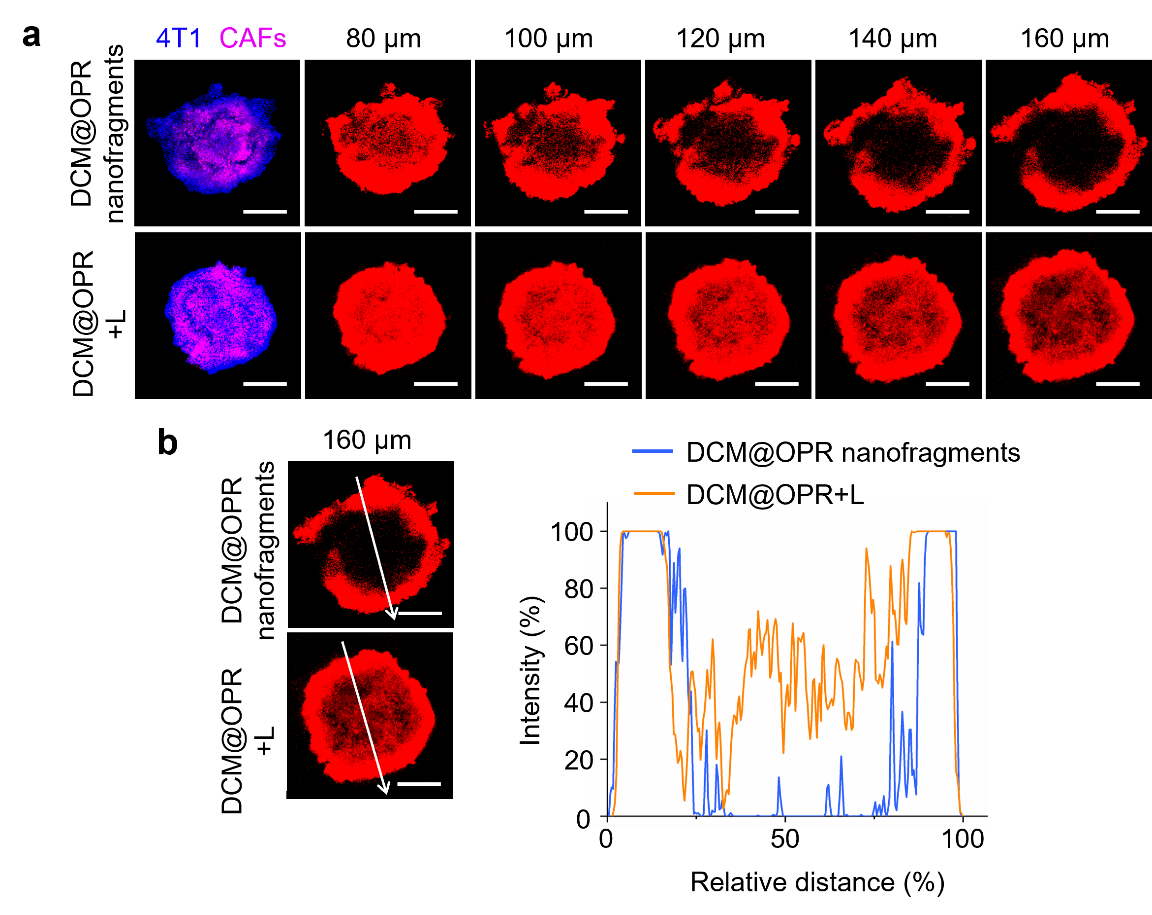


**Figure. S19.** **a**) Deep penetration capacity of DCM@OPR nanofragments and DCM@OPR+L groups in heterospheroids. **b**) Corresponding fluorescence intensity along the white line at 160 μm scanning depth in heterospheroids. Scale bars: 100 μm.


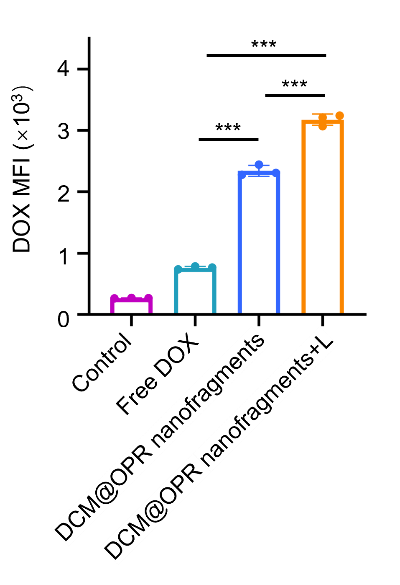


**Figure. S20.** The cellular uptake of free DOX and DCM@OPR nanofragments and DCM@OPR nanofragments with laser irradiation. Data are presented as mean ± SD (n = 3). **p* < 0.05, ***p* < 0.01, ****p* < 0.001.


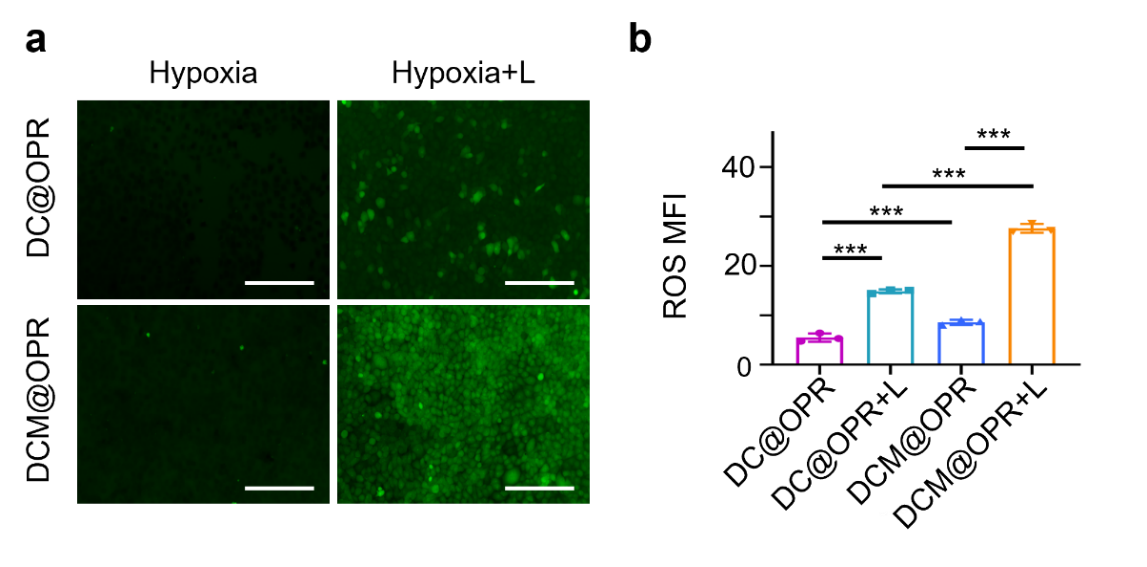


**Figure. S21.** ROS generation under hypoxia condition. **a**) Fluorescent images of ROS generation using the fluorescent probe DCFH-DA in 4T1 cells. Scale bars: 300 μm. **b**) The MFI of ROS in 4T1 cells. Data are presented as mean ± SD (n = 3). **p* < 0.05, ***p* < 0.01, ****p* < 0.001.


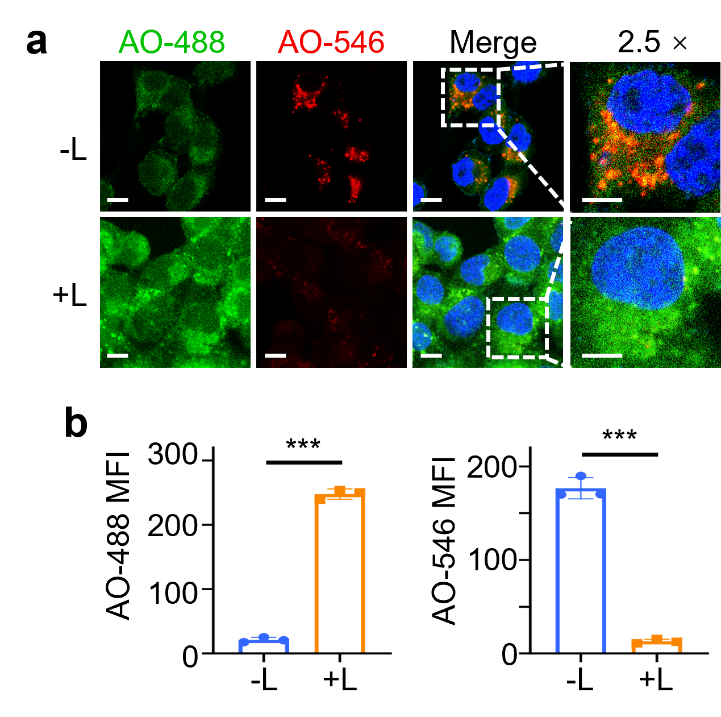


**Figure.S22. a**) Immunofluorescent analysis of the AO-staining using CLSM before and after laser irradiation. Scale bars: 10 μm. **b**) MFI statistics of AO-488 and AO-546. Data are presented as mean ± SD (n = 3). **p* < 0.05, ***p* < 0.01, ****p* < 0.001.


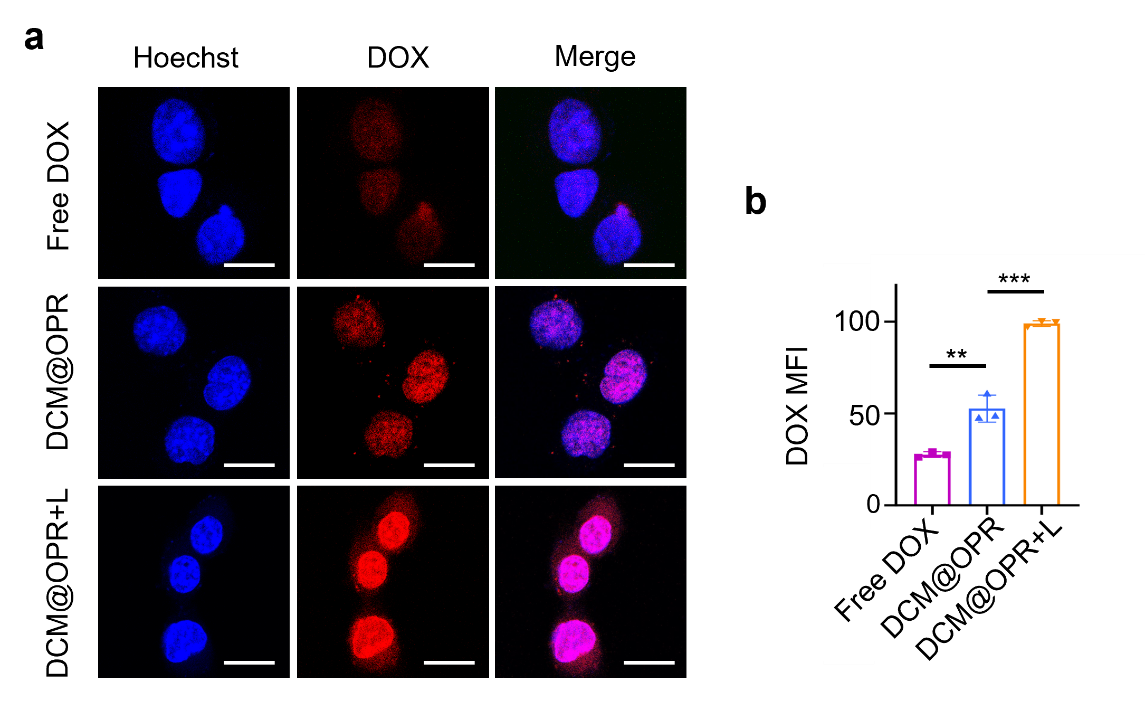


**Figure. S23.** Cellular internalization of DOX in 4T1 cells. **a**) CLSM images of free DOX, DCM@OPR and DCM@OPR with laser irradiation (DCM@OPR+L) groups (DOX equivalent: 2.5 μg/mL) in 4T1 cells. Scale bars: 10 μm. **b**) The MFI of DOX in 4T1 cells. Data are presented as mean ± SD (n = 3). **p* < 0.05, ***p* < 0.01, ****p* < 0.001.


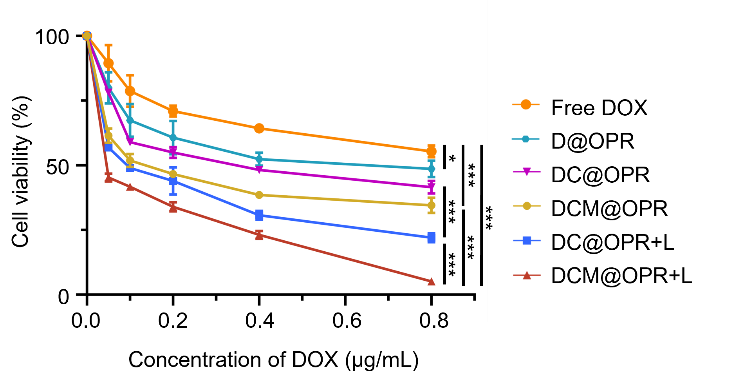


**Figure. S24.** The inhibitory proliferation effect of different groups on 4T1 cells determined by MTT assay. Data are presented as mean ± SD (n = 3). **p* < 0.05, ***p* < 0.01, ****p* < 0.001.


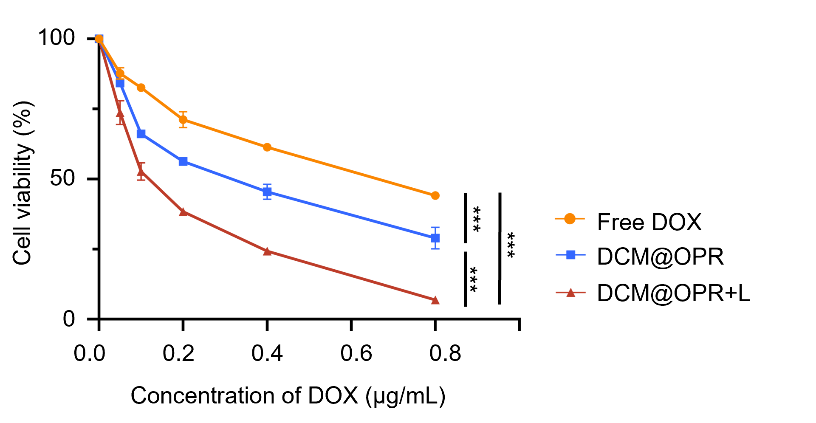


**Figure. S25.** The inhibitory proliferation effect of different groups on MDA-MB-231 cells determined by MTT assay. Data are presented as mean ± SD (n=3). **p* < 0.05, ***p* < 0.01, ****p* < 0.001.


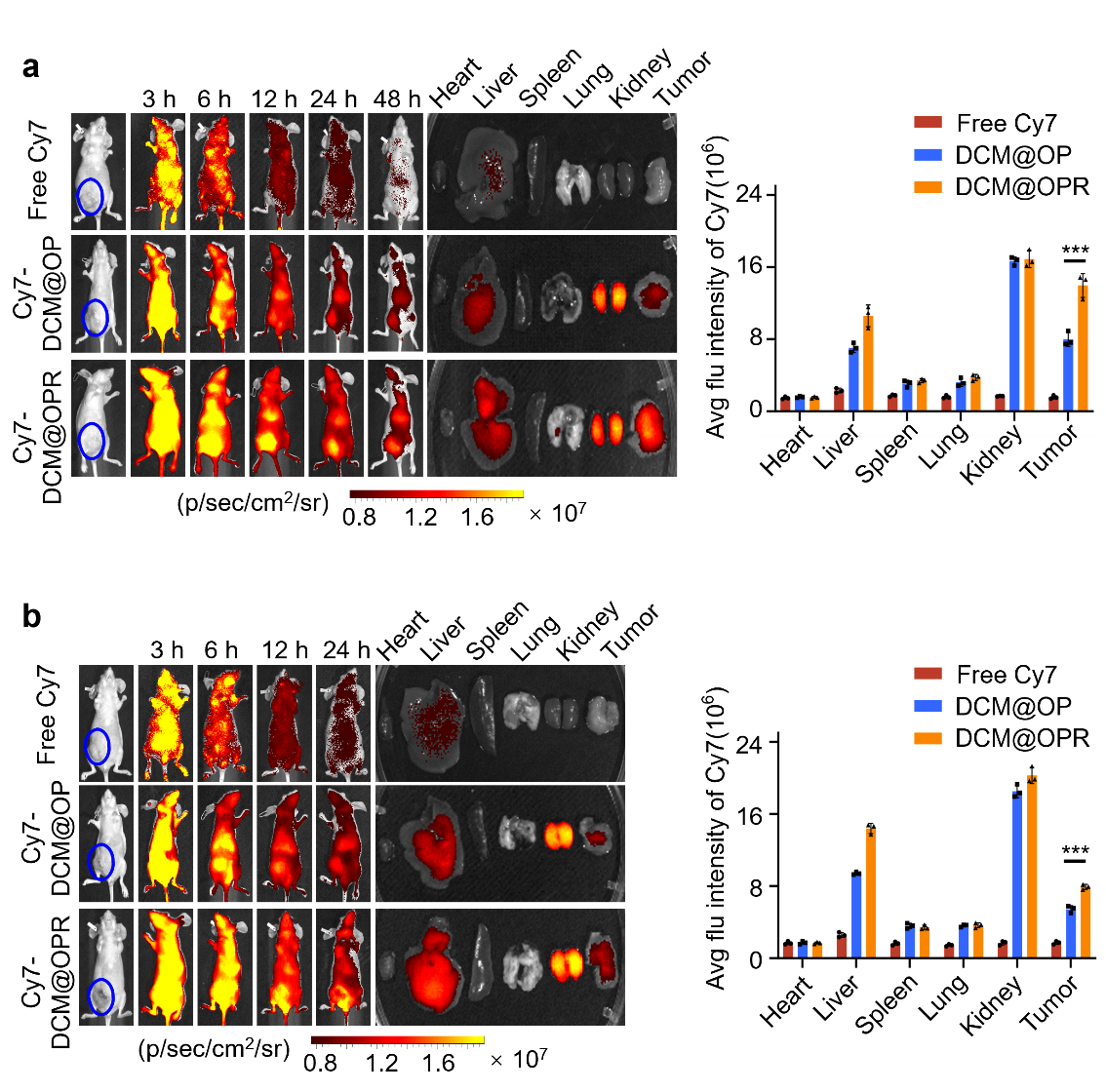


**Figure. S26.** Biodistribution and tumor targeting of photodynamic gel-bombs (DCM@OPR) *in vivo*. Biodistribution of free Cy7, Cy7-DCM@OP and Cy7-DCM@OPR in 4T1 *in situ* tumor-bearing Balb/c nude mice at different time points over **a**) 48 h or **b**) 24 h and average fluorescence intensity of *ex vivo* organs and tumors in different groups. Data are presented as mean ± SD (n = 3). **p* < 0.05, ***p* < 0.01, ****p* < 0.001.


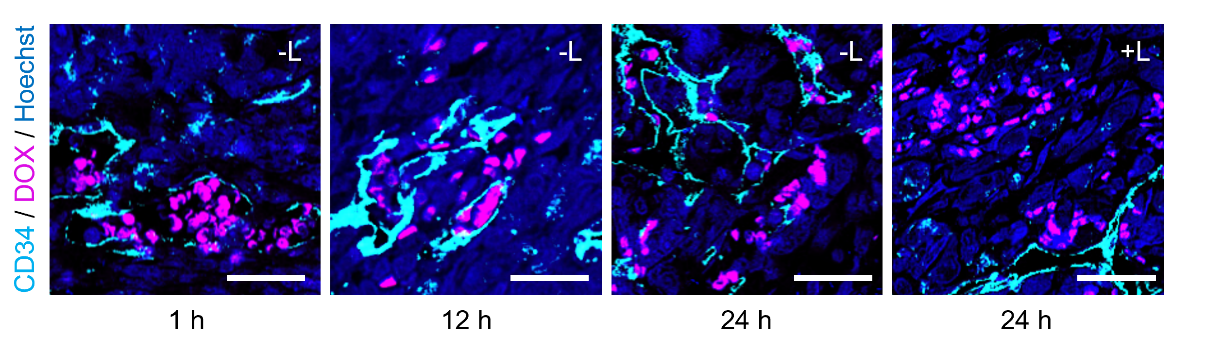


**Figure. S27.** The confocal laser scanning microscope images of DCM@OPR intratumoral distribution after intravenous administration in 4T1-bearing mice at different times. Endothelial cells stained with CD34 antibody (cyan), nucleus stained with Hoechst 33342 (blue) and DCM@OPR marked with DOX (purple). Scale bars: 40 μm.


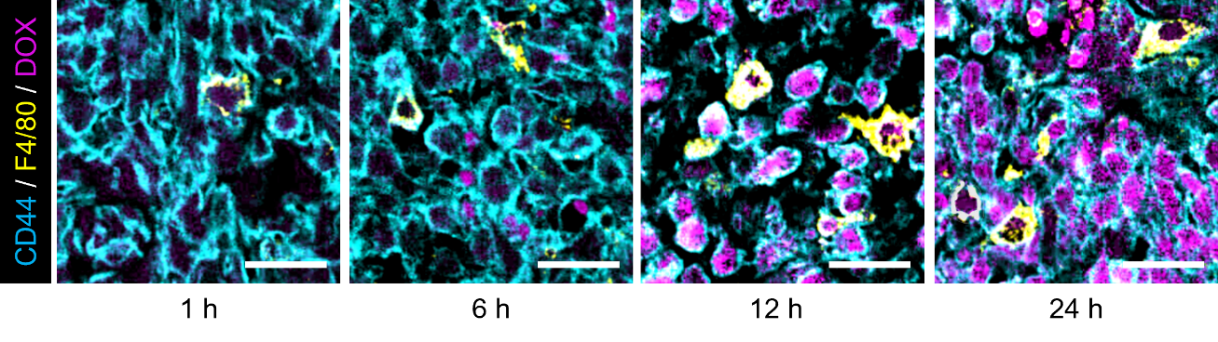


**Figure. S28.** The intratumoral distribution of DCM@OPR after intravenous administration in 4T1-bearing mice at different times. 4T1 cells marked with CD44 antibody (cyan), macrophages with F4/80 antibody (yellow) and DCM@OPR marked with DOX (purple). Scale bars: 20 μm.


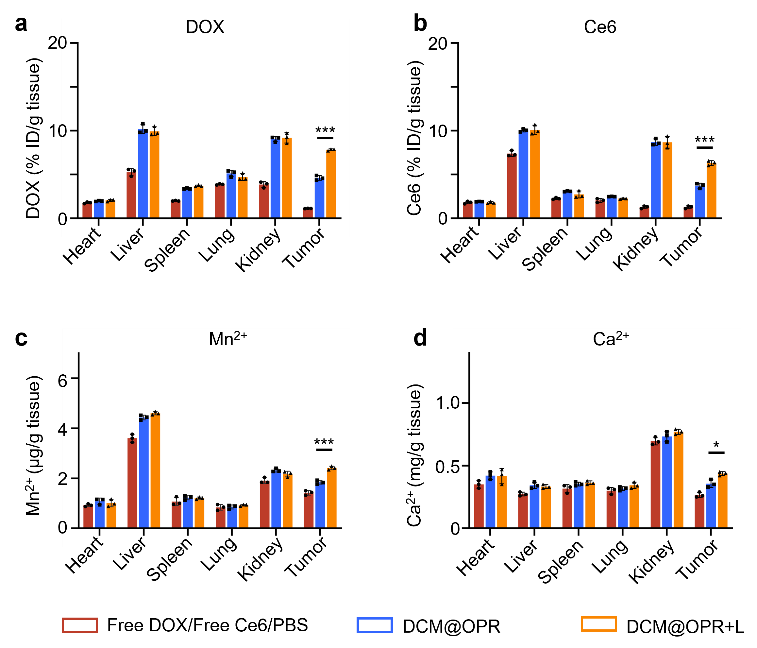


**Figure. S29.** Drug accumulation of **a**) DOX, **b**) Ce6, **c**) Mn^2+^ and **d**) Ca^2+^ in major organs and tumors of 4T1 *in situ* tumor-bearing Balb/c mice after treatment of free DOX/free Ce6/PBS, DCM@OPR, DCM@OPR with laser irradiation (DCM@OPR+L), respectively. Data are presented as mean ± SD (n = 3). **p* < 0.05, ***p* < 0.01, ****p* < 0.001.


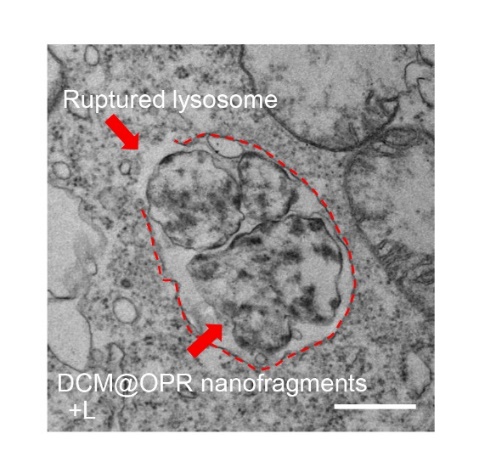


**Figure. S30.** TEM image of DCM@OPR nanofragments in 4T1 tumor cell after laser irradiation. The result suggested that upon exposure to laser irradiation, photodynamic gel-bombs (DCM@OPR) disintegrates into nanofragments. Meanwhile, the photodynamic-triggered explosive energy damages the lysosomal membrane. Scale bar: 500 nm.


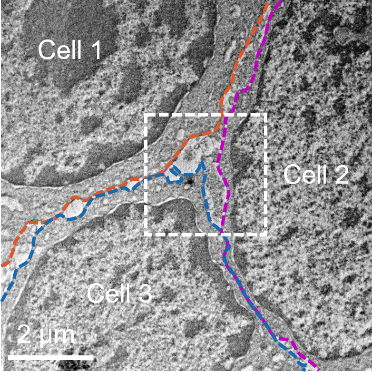


**Figure. S31.** TEM image of the penetration of DCM@OPR nanofragments through gap leakage among tumor cells. Scale bar: 2 μm.


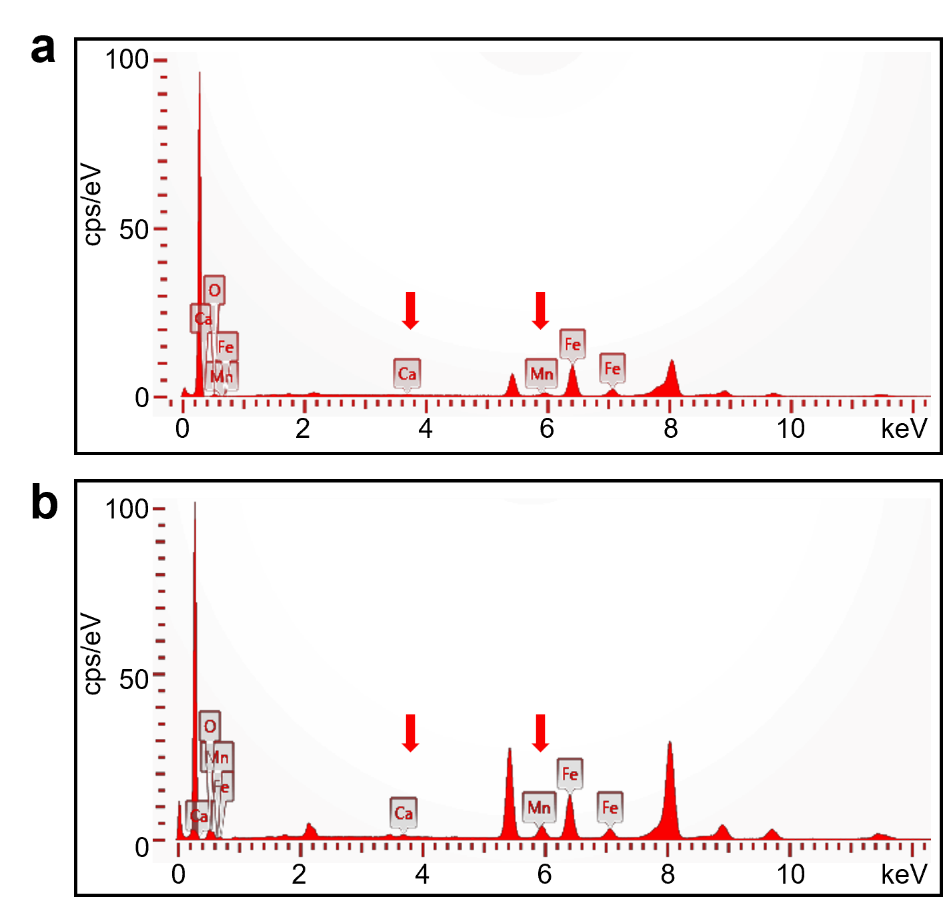


**Figure. S32.** Elemental analysis of 4T1 tumor tissue TEM images **a**) before and **b**) after laser irradiation using EDS.


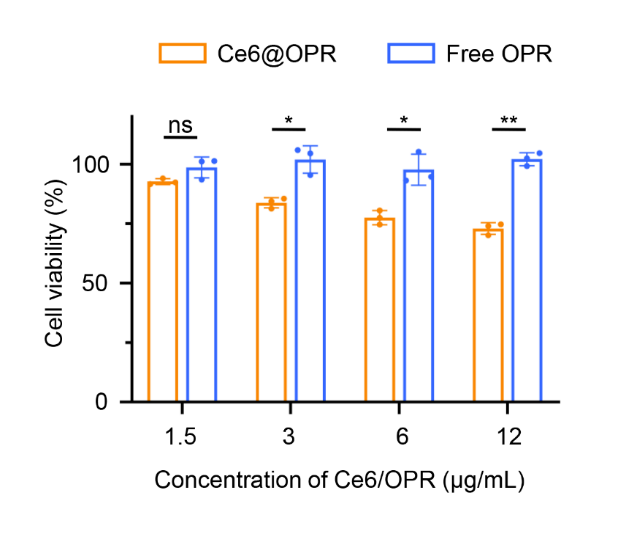


**Figure. S33.** The cell viability of Ce6@OPR and free OPR. Data are presented as mean ± SD (n = 3). **p* < 0.05, ***p* < 0.01, ****p* < 0.001, ns means no statistical significance.


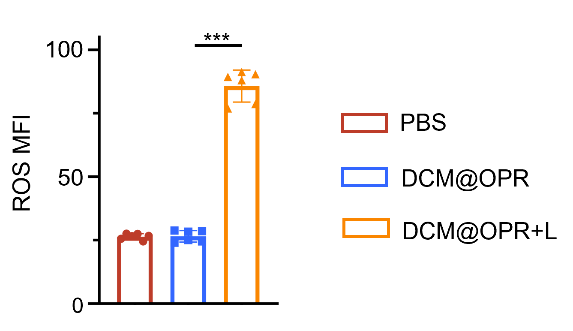


**Figure. S34.** Flow cytometry detection of ROS in tumor tissues. Data are presented as mean ± SD (n = 6). **p* < 0.05, ***p* < 0.01, ****p* < 0.001.


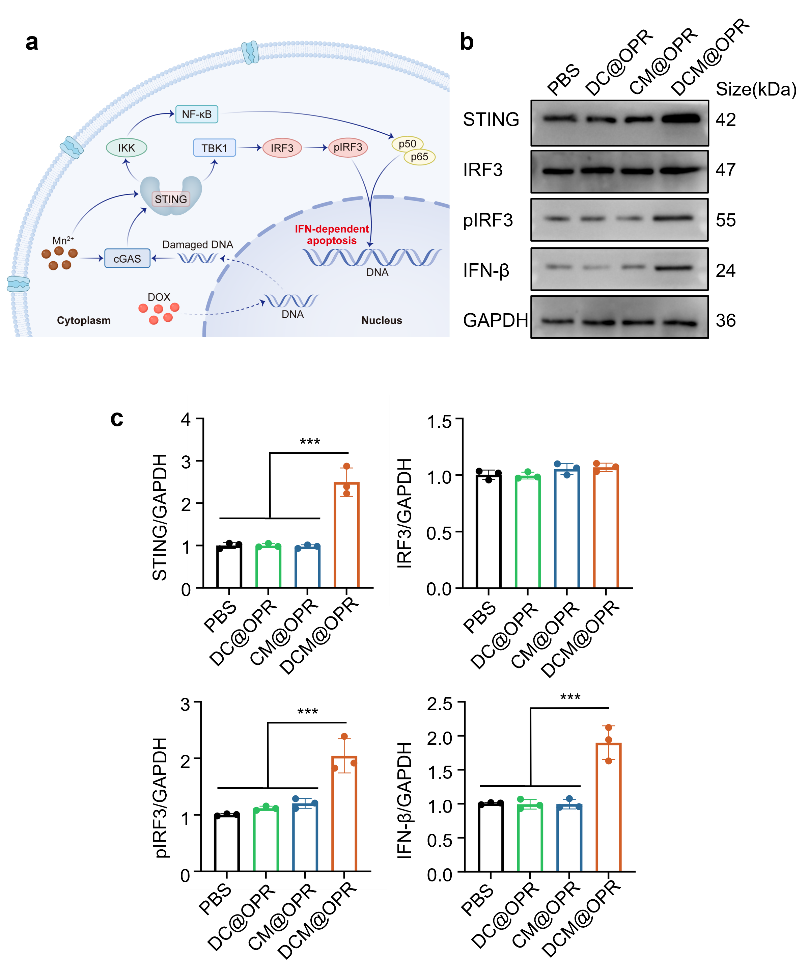


**Figure. S35.** **a**) Schematic diagram of the cGAS/STING signaling pathway. **b**) WB analysis of 4T1 tumor protein levels after treatment with PBS, DC@OPR, CM@OPR and DCM@OPR. **c**) Quantification of the expression levels of STING, IRF3, pIRF3 and IFN-β protein. Data are presented as mean ± SD (n = 3). **p* < 0.05, ***p* < 0.01, ****p* < 0.001, unmarked means no statistical significance.


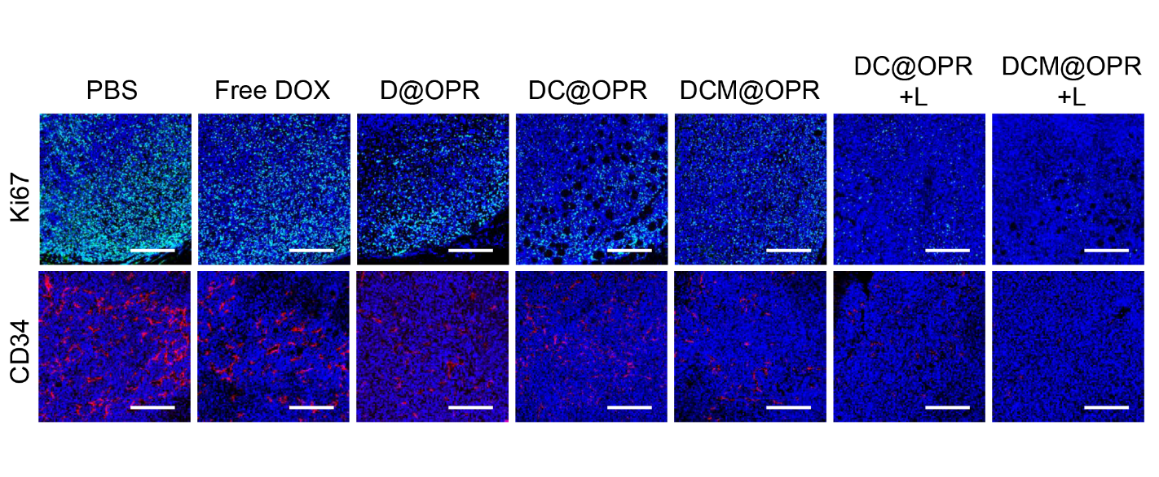


**Figure. S36.** IF of 4T1 tumors from different treatment groups for cell proliferation using Ki67 and for endothelial vessels using CD34. Scale bars: 200 μm.


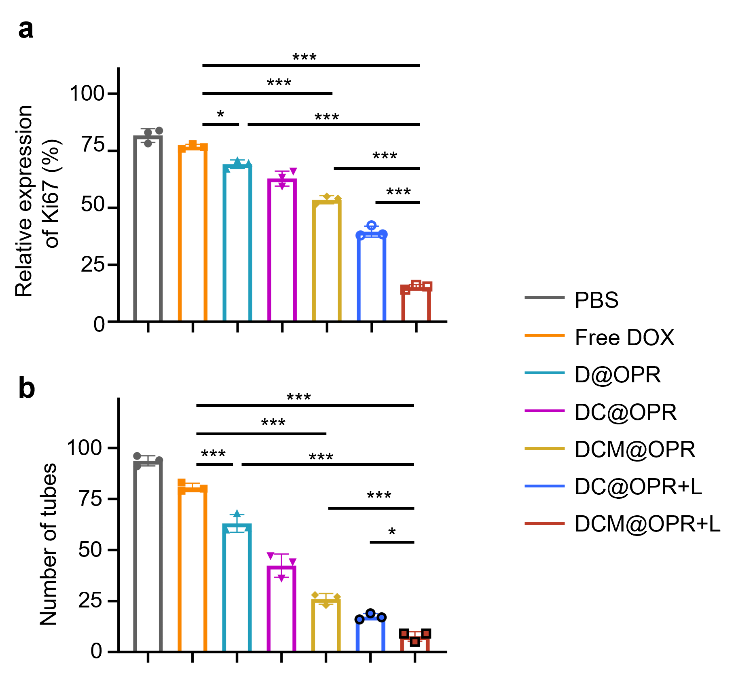


**Figure. S37.** The IHC quantification results of **a**) Ki67 and **b**) CD34 in each treatment group.

Data are presented as mean ± SD (n = 3). **p* < 0.05, ***p* < 0.01, ****p* < 0.001.


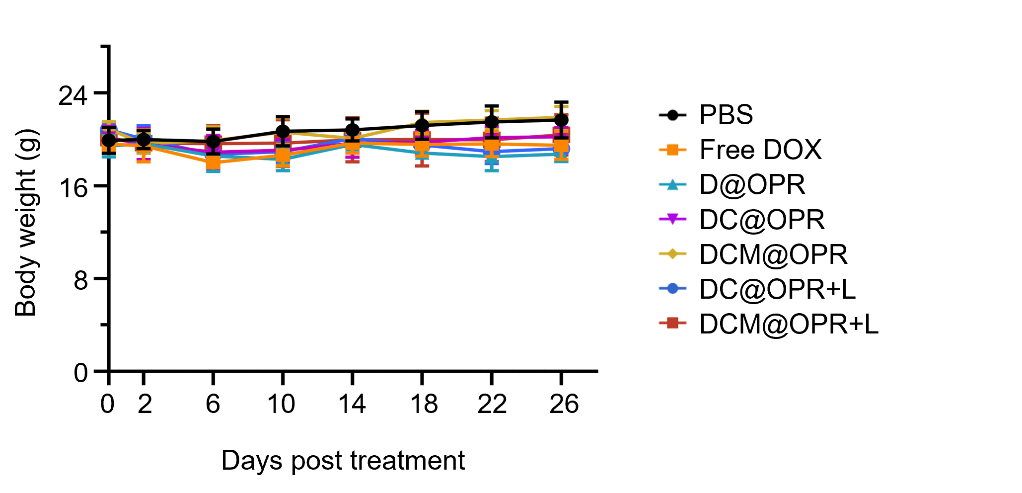


**Figure. S38.** The body weight of mice over 26 day period in each treatment group. Results are expressed as mean ± SD (n = 6).


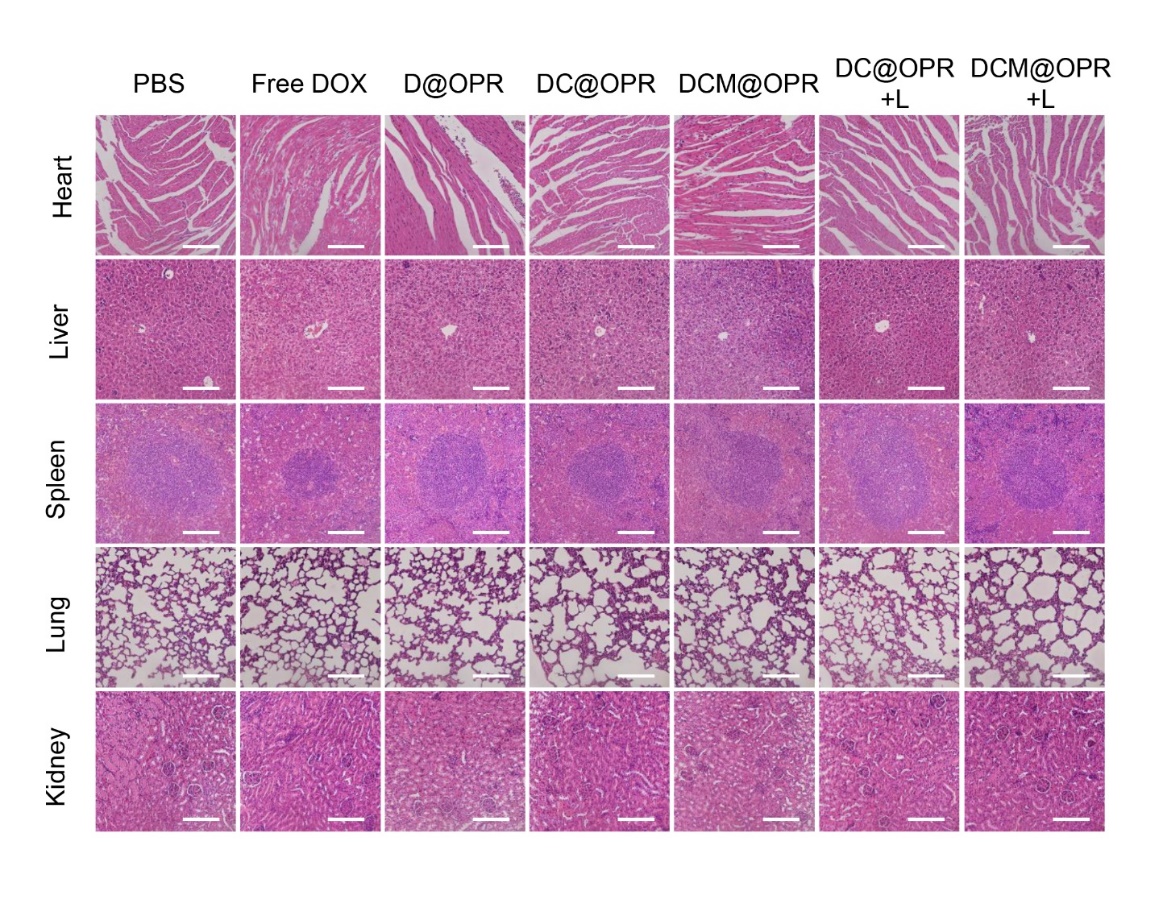


**Figure. S39.** Optical images of H&E staining of the main organs of 4T1 *in situ* tumor-bearing Balb/c mice in each treatment group. Scale bars: 100 μm.


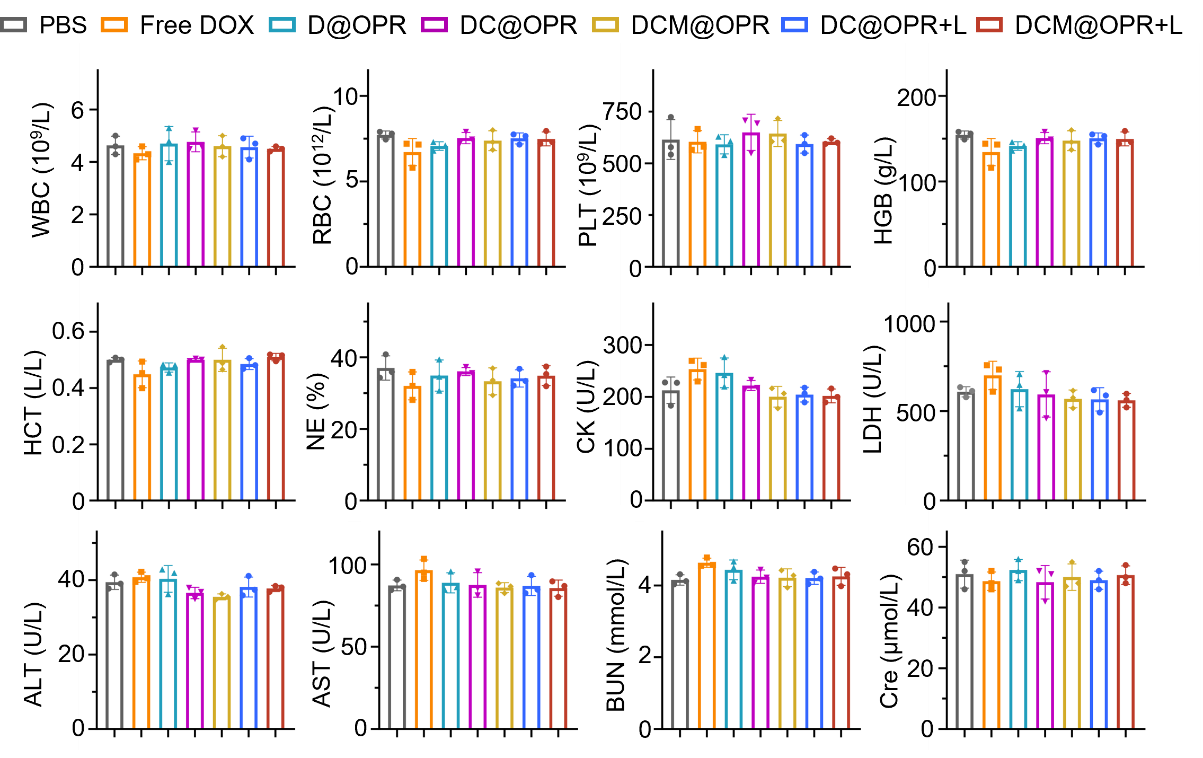


**Figure. S40.** The routine blood tests (including WBC count, RBC count, PLT, HGB, HCT, NE%), and serological test (including CK, LDH, ALT, AST, BUN and Cre) in each treatment group (2.5 mg DOX equivalent/kg, 1.5 mg Ce6 equivalent/kg). Results are expressed as mean ± SD (n = 3). *p* -values were analyzed by one-way ANOVA with Bonferroni correction, unmarked means no statistical significance.


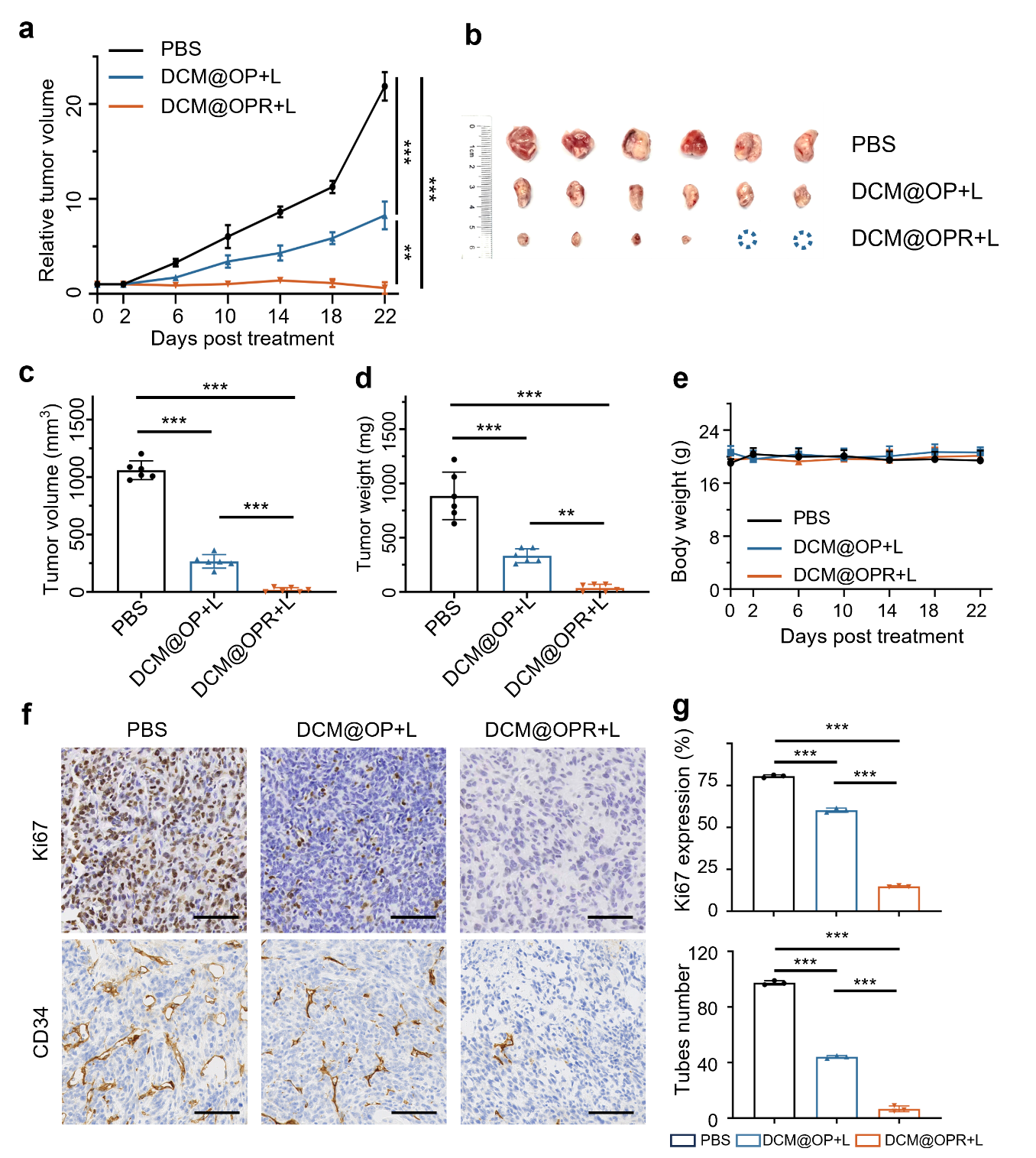


**Figure. S41.** ***In vivo* anti-tumor efficacy of DCM@OP and DCM@OPR in 4T1 *in situ* tumor-bearing Balb/c mice.** **a**) Relative tumor volumes over time in mice from PBS, DCM@OP+L and DCM@OPR+L groups. n = 6 per group. **b**) Excised tumors from mice in each group. **c**) Excised tumor volume in 3 different groups at the end of the experiment. n = 6 per group. **d**) The excised tumor volumes of mice from each group. n = 6 per group. **e**) The excised tumor weights of mice from each group. n = 6 per group. **f**) IHC staining of 4T1 tumors from each group. Scale bars: 100 μm. n = 3 per group. **g**) The corresponding quantitative results of IHC for Ki67 and CD34 in each group. n = 3 per group. Data are presented as mean ± SD. **p* < 0.05, ***p* < 0.01, ****p* < 0.001.


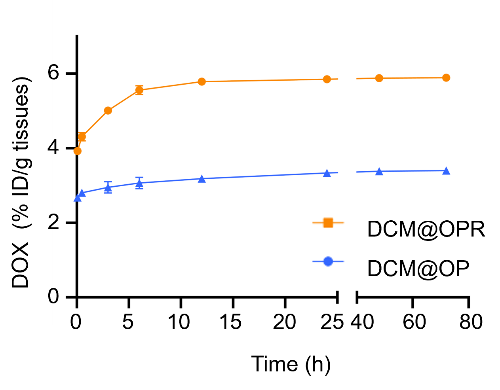


**Figure. S42.** DOX concentration in tumor tissues were detected at different time points after DCM@OP and DCM@OPR were injected into mice via tail vein administration. Results are expressed as mean ± SD (n = 3).


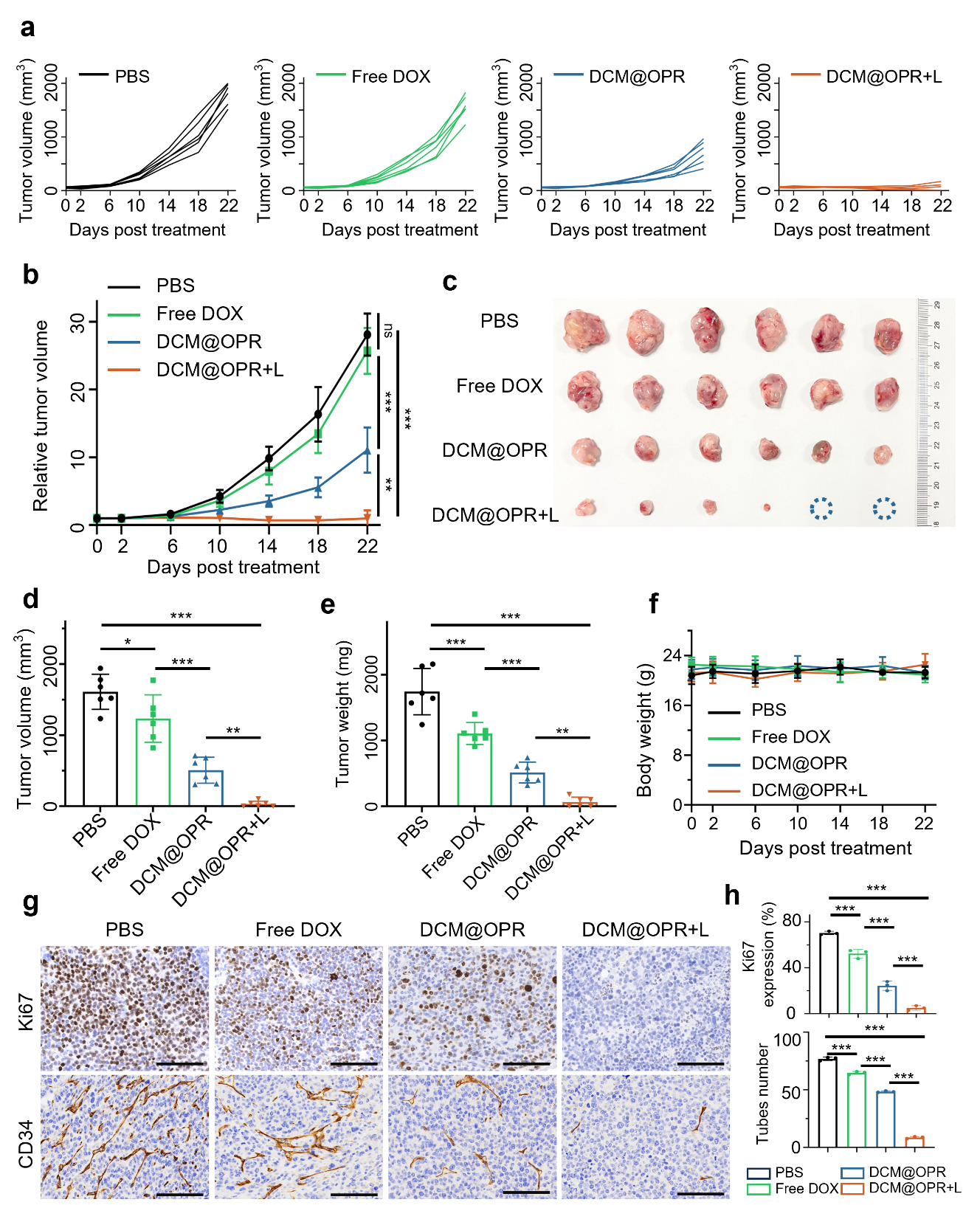


**Figure. S43. *In vivo* evaluation of the photodynamic gel-bombs (DCM@OPR) in the *in situ* MDA-MB-231 breast cancer xenograft model.** **a**) Tumor growth curves of the MDA-MB-231 tumor bearing mice during treatment. n = 6 per group. **b**) Relative tumor volumes over time in MDA-MB-231 tumor bearing mice from different groups during treatment. n = 6 per group. **c**) Photographs of tumors taken from different groups of mice after treatment. **d**) Excised tumor volume of different treatment groups. n = 6 per group. **e**) Excised tumor weight of different treatment groups. n = 6 per group. **f**) Changes of average body weights from mice during different treatments. n = 6 per group. **g**) Ki67 and CD34 IHC staining of the collected tumors from different groups. n = 3 per group. Scale bars: 100 μm. **h**) The IHC corresponding quantitative results of Ki67 and CD34 in different groups. n = 3 per group. Data are presented as mean ± SD. **p* < 0.05, ***p* < 0.01, ****p* < 0.001.


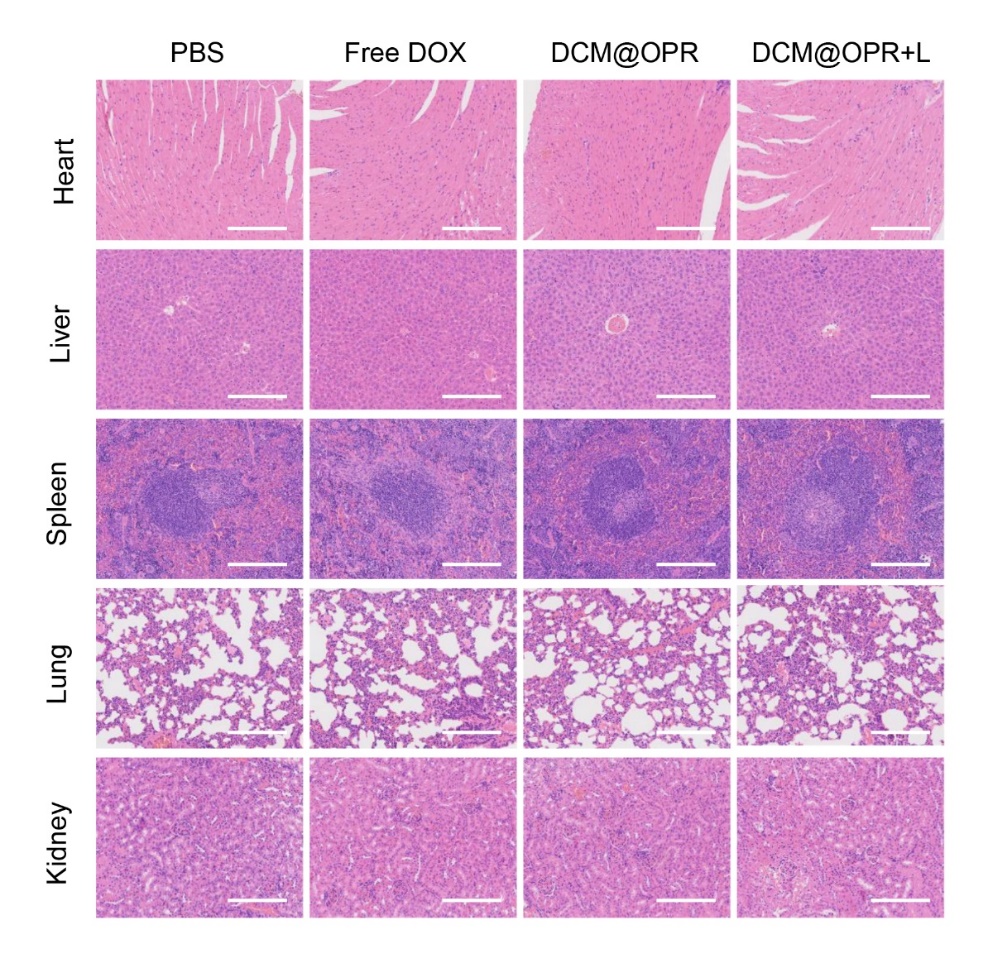


**Figure. S44.** Optical images of H&E staining of the main organs of MDA-MB-231 *in situ* tumor-bearing Balb/c nude mice treated with PBS, free DOX, DCM@OPR, and DCM@OPR+L. Scale bars: 200 μm.


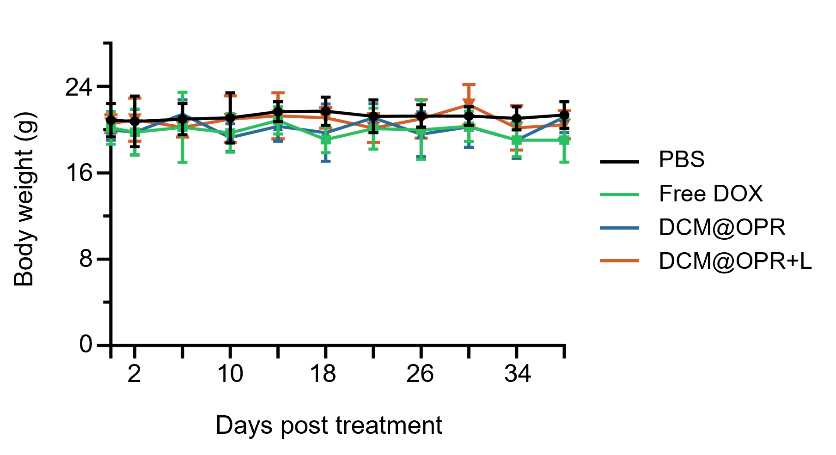


**Figure. S45.** The body weight of PDX mice over 38 day period in each treatment group. Results are expressed as mean ± SD (n = 6).


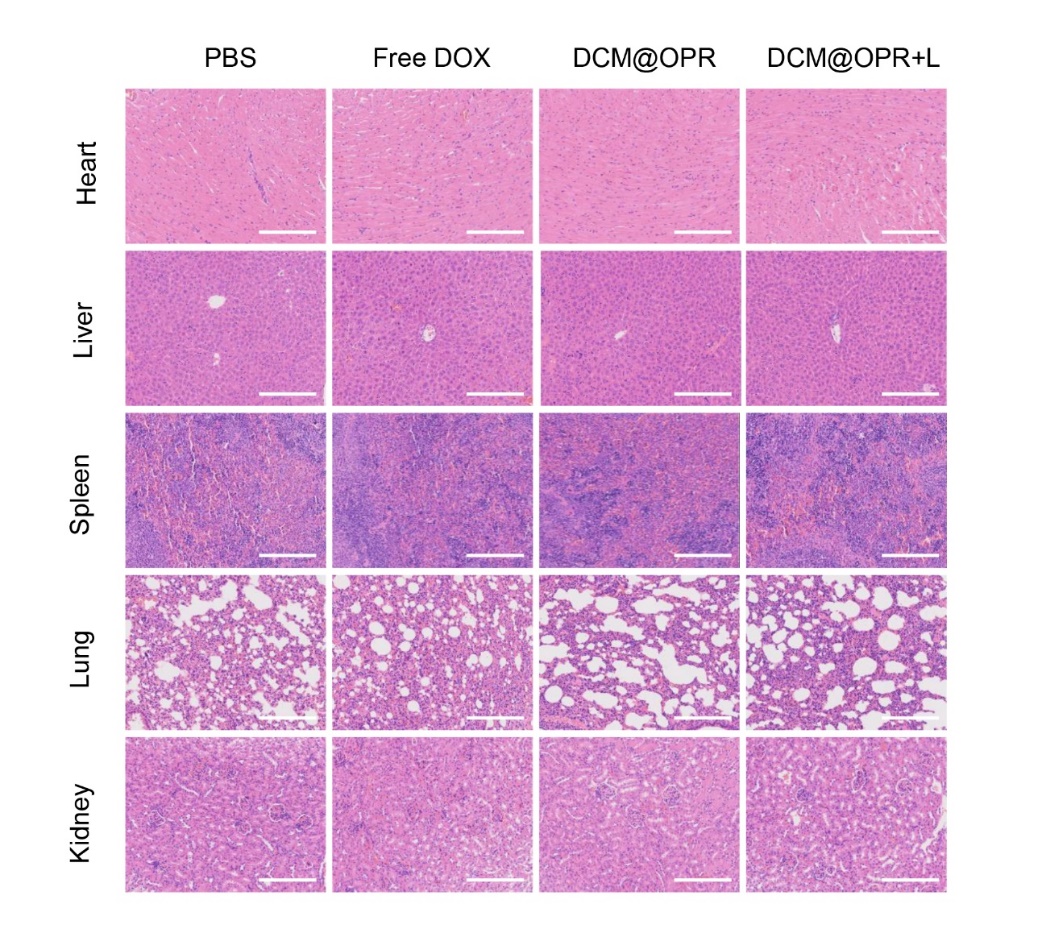


**Figure. S46.** Optical images of H&E staining of the main organs of PDX TNBC models mice treated with PBS, free DOX, DCM@OPR, and DCM@OPR+L. Scale bars: 200 μm.

**Table S1.** The EE% and LE% of DOX, Ce6 and MnO_2_ within the photodynamic gel-bombs (DCM@OPR).

| Entry | Encapsulation Efficiency (EE%) | Loading efficiency (LE%) |
| --- | --- | --- |
| DOX | 83.43 ± 1.21 | 23.09 ± 0.45 |
| Ce6 | 51.37 ± 0.80 | 17.02 ± 0.16 |
| MnO_2_ | 49.87 ± 3.70 | 13.70 ± 0.37 |

Data were presented as means ± SD (n = 3).

**Table S2.** The table of molecular weight.

| Entry | Mn | Mw | Mw/Mn |
| --- | --- | --- | --- |
| DCM@OPR | 34922 | 44748 | 1.28 |
| DCM@OPR nanofragments | 16362 | 17660 | 1.08 |

Mn: Number-Average Molecular Weight; Mw: Weight-Average Molecular Weight

**Table S3.** The EE% of DOX, Ce6 and MnO_2_ within the nanofragments.

| Entry | Encapsulation Efficiency (EE%) |
| --- | --- |
| DOX | 79.83 ± 2.03 |
| Ce6 | 45.58 ± 1.43 |
| MnO_2_ | 47.62 ± 2.92 |

Data were presented as means ± SD (n = 3).

**Table S4.** The IC50 of D@OPR and DCM@OPR+L

| Entry | IC50 ± SD (concentration of DOX, ng/mL) |
| --- | --- |
| D@OPR | 712.85 ± 14.41 |
| DCM@OPR+L | 27.67 ± 2.26 |

**Table S5.** The IC50 of CM@OPR+L and DCM@OPR+L

| Entry | IC50 ± SD (concentration of Ce6, ng/mL) |
| --- | --- |
| CM@OPR+L | 73.55 ± 3.15 |
| DCM@OPR+L | 16.60 ± 1.36 |

**Table S6.** The weight percent (Wt%) of calcium (Ca) and manganese (Mn) determined by EDS

| Element | -L | +L |
| --- | --- | --- |
| Ca | 0.98 | 1.94 |
| Mn | 3.98 | 11.04 |

-L: without laser irradiation”; +L: with laser irradiation
